# Mechanisms and Therapeutic Potential of Nutritional Immunity

**DOI:** 10.3390/pathogens15020176

**Published:** 2026-02-05

**Authors:** Charles Egede Ugwu, Olalekan Chris Akinsulie, Toyin Florence Ayandokun, Favour Akinfemi Ajibade, Sammuel Shahzad, Victor Ayodele Aliyu, Moyinoluwa Joshua Oladoye, Ibrahim Idris, Kingsley Ogochukwu Obasi, Joel Kosisochukwu Edeh, Al-Amin Adebare Olojede, Chizaram Blessing Ukauwa, Muhammad Ipoola Adeyemi, Charity Chinonso Ugwu, Lilian Chizobam Ugorji

**Affiliations:** 1 Paul G. Allen School for Global Health, College of Veterinary Medicine, Washington State University, Pullman, WA 99164, USA; 2 Department of Veterinary Microbiology and Pathology, College of Veterinary Medicine, Washington State University, Pullman, WA 99164, USA; 3 Department of Chemistry, College of Arts and Sciences, Washington State University, Pullman, WA 99164, USA; 4 Department of Microbiology and Molecular Genetics, The Robert Larner, M.D. College of Medicine, University of Vermont, Burlington, VT 05405, USA; 5 School of Molecular Biosciences, College of Veterinary Medicine, Washington State University, Pullman, WA 99164, USA; 6 School of Veterinary Medicine, Texas Tech University, Amarillo, TX 79106, USA; 7 Department of Biomedical Sciences, University of Wolverhampton, Wolverhampton WV1 1LY, West Midlands, UK; 8 Department of Medical Laboratory Sciences, University of Nigeria, Nsukka 410001, Enugu State, Nigeria; 9 Faculty of Veterinary Medicine, University of Ibadan, Ibadan 200001, Oyo State, Nigeria; 10 Faculty of Veterinary Medicine, University of Abuja, Abuja 902101, Federal Capital Territory, Nigeria; 11 Department of Biological Sciences, University of Bergen, 5007 Bergen, Vestland County, Norway; 12 Faculty of Pharmaceutical Sciences, Nnamdi Azikiwe University, Awka 420110, Anambra State, Nigeria; 13 Department of Microbiology, Imo State University, Owerri 460108, Imo State, Nigeria

**Keywords:** nutritional immunity, host–pathogen interactions, metal sequestration, immunometabolism, pathogen adaptation, host-directed therapeutics, antimicrobial resistance

## Abstract

Nutritional immunity is a major facet of host defense, wherein the host immune system strategically limits pathogen access to critical nutrients, including iron, zinc, vitamins, lipids, and amino acids, to repress microbial proliferation and virulence. This review provides a comprehensive synthesis of the molecular mechanisms that power nutrient immunity, including metal homeostasis, nutrient competition, transporter modulation, hormonal regulation, and direct antimicrobial actions. We examine nutrient-specific strategies employed by the host, such as iron-withholding mechanisms, vitamin deprivation, and copper-mediated toxicity. We also explore how diverse pathogens, including extracellular, intracellular, and eukaryotic pathogens, adapt to these hostile nutritional landscapes through siderophore diversification, regulatory integration, and metabolic rewiring. Comparative genomic analyses reveal convergent evolution in nutrient acquisition systems, illuminating the dynamic arms race between host restriction and microbial evasion. We examine the immunological mechanisms that regulate nutritional immunity. Further, we discuss the translational potential of nutritional immunity, cutting across nutrient-based therapies, host-directed interventions, and emerging diagnostic biomarkers. Finally, we suggest future directions that synergize nutritional immunity with microbiome ecology, global malnutrition, and personalized medicine. By elucidating the interconnection between metabolism and immunity, this review highlights the therapeutic promise of starving or toxifying the pathogen to save the host.

## 1. Introduction

Infectious diseases constitute a major public health concern across the globe [1]. The situation is further aggravated by the increasing emergence of antimicrobial resistance and inadequate development of new antibiotics [2,3]. At the heart of every infection lies a fierce competition for nutrients between the host and an invading pathogen [4]. Pathogens, including bacteria, fungi, and protozoa, need access to essential elements such as zinc, iron, manganese, amino acids, and vitamins to uphold structural integrity, power replication, and initiate their virulence programs [5,6]. In response, the host deploys a repertoire of defense mechanisms collectively known as nutritional immunity, which are strategies that limit microbial access to these resources while maintaining host metabolic homeostasis [7]. Interestingly, this nutrient tug-of-war is not just a passive consequence of infection but rather a dynamic and evolutionarily conserved interface between metabolism and immunity. For example, iron sequestration via transferrin, lactoferrin, and hepcidin is tightly controlled to restrict microbial proliferation while minimizing oxidative stress [8]. Similarly, calprotectin-mediated zinc and manganese chelation hampers bacterial enzymatic function and growth [9]. These host strategies are opposed by microbial adaptations, including siderophore production, metal transporter upregulation, and metabolic rewiring, underlining the complexity of host–pathogen nutrient interactions [10]. A snapshot of major nutrients in infection at the host–pathogen interface is offered in Table 1. The table presents the physiological role of these nutrients in both host and pathogen, pathogen strategies for acquiring these nutrients from the host, and some infection contexts. Furthermore, nutritional immunity is increasingly recognized as a key pillar of innate and adaptive defense. It borders cytokine signaling, hormonal regulation, and cellular immunity, determining the outcome of infections across different anatomical niches [5,11]. For instance, macrophages regulate phagosomal nutrient availability through natural resistance-associated macrophage protein 1 (NRAMP1)-mediated metal efflux and autophagy-linked amino acid deprivation [12,13]. In parallel, systemic responses such as hypoferremia and altered vitamin metabolism reveal the host’s attempt to starve pathogens while maintaining immune function [14]. Notably, the concept of nutritional immunity is not only true within the context of bacterial infections. Eukaryotic pathogens such as *Plasmodium falciparum*, *Leishmania* spp., and *Candida albicans* also exhibit complex nutrient acquisition systems that allow immune evasion and persistence [6,15]. Importantly, nutritional immunity must also be understood within a broader global health context, where baseline nutritional status, food security, and environmental change shape host susceptibility to infection. For instance, in regions burdened by malnutrition, deficiencies in iron, zinc, protein, and vitamins profoundly alter immune competence and the host’s ability to restrict pathogen access to essential nutrients [16,17]. These interactions generate epidemiological trade-offs, such as the paradoxical relationship between iron deficiency and malaria risk, that complicate public health interventions [14]. As climate change, urbanization, and dietary transitions reshape pathogen ecology and nutrient availability worldwide, nutritional immunity emerges not only as a mechanistic pillar of host defense but also as a unifying framework that interlinks immunology, metabolism, microbiome composition, environmental factors, and global health [18,19,20,21]. This review, whose rationale is presented in Supplementary Material aims to provide a comprehensive synthesis of the mechanisms, pathogen adaptation, and therapeutic implications of nutritional immunity. We begin by detailing the molecular and cellular processes through which the host limits pathogen access to critical nutrients, including iron restriction, copper toxicity, and glucose deprivation, and examine how diverse pathogens adapt to these pressures. We offer comparative genomic insights that reveal evolutionary patterns in nutrient acquisition. We discuss the immunological mechanisms that control nutritional immunity and present translational perspectives that highlight emerging therapies and diagnostics rooted in nutritional immunity. We conclude by outlining future directions that would interlink nutritional immunity with microbiome science, personalized medicine, and global health.

## 2. Mechanisms of Nutritional Immunity and Pathogen Adaptation

**Figure 1:**
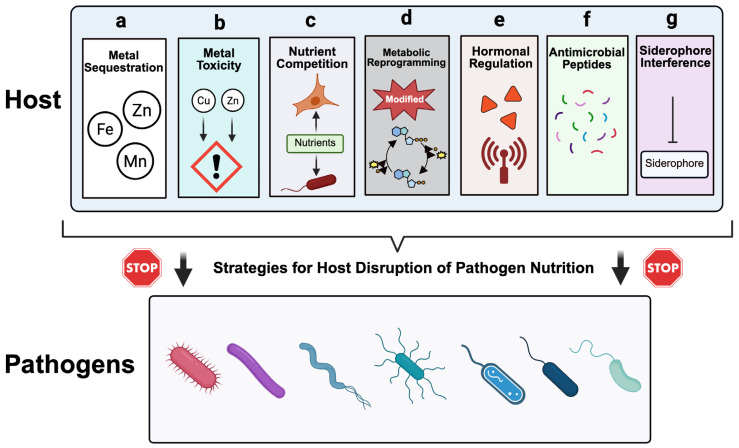
Schematic illustration of nutritional immunity mechanisms. The various strategies used by the host to disrupt pathogen nutrition include: (**a**), metal sequestration; the host sequesters essential metals like iron (Fe), zinc (Zn), and manganese (Mn) that pathogens need to grow and multiply. (**b**), metal toxicity; host intentionally delivers toxic levels of certain transition metals into pathogen-containing compartments, which overwhelms microbial detoxification systems, and may have pathogens incorporate incorrect metals into their enzymes, such as copper (Cu) instead of iron, leading to dysfunctional proteins and impaired microbial survival. (**c**), nutrient competition; host cells and immune cells compete with pathogens for key nutrients like glucose, lipids, and amino acids. (**d**), metabolic reprogramming; the host alters its own metabolism during infection to reduce the availability of nutrients like glucose and amino acids to pathogens. (**e**), hormonal regulation; inflammatory cytokines and hormones like hepcidin regulate iron homeostasis by decreasing iron absorption and trapping iron in storage sites. (**f**), production of antimicrobial peptides; molecules like calprotectin bind and sequester multiple metals, including zinc and manganese, but also have direct antimicrobial effects. (**g**), siderophore interference; hosts produce proteins like lipocalin-2 that bind bacterial siderophores, which are iron-scavenging molecules, preventing pathogens from retrieving iron.

The concept of nutritional immunity majorly encompasses a suite of host strategies designed to restrict microbial access to essential nutrients within the host milieu, thereby impairing pathogen proliferation and virulence. These mechanisms, which are, however, multifaceted and evolving, are summarized in Figure 1 and Table 2 and will be discussed extensively in this section. Concisely, the mechanisms of nutritional immunity involve metal sequestration, metal toxicity, nutrient competition, metabolic reprogramming, hormonal regulation, siderophore interference, and direct antimicrobial actions. Together, they constitute a dynamic and context-dependent defense system that integrates innate and adaptive immune responses. Notably, in humans, nutritional immunity is executed predominantly through dynamic redistribution and compartmentalization of nutrients, rather than through global depletion of total body stores. For example, following immune activation as a result of infection, the acute-phase response, driven largely by inflammatory cytokines such as interleukin-6 (IL-6), orchestrates rapid and coordinated changes in nutrient trafficking across tissues and cellular compartments, thereby decreasing pathogen access to critical metabolites while preserving host metabolic integrity [11,31,32]. This spatial regulation is increasingly recognized as a defining feature of nutritional immunity in human infection and inflammation [33]. A major component of this process is the differential regulation of positive and negative acute-phase proteins. For instance, while positive acute-phase proteins such as hepcidin, ferritin, and lipocalin-2 promote intracellular sequestration and restrict metal export, negative acute-phase proteins, including albumin and transferrin, diminish in circulation during inflammation, facilitating the relocalization of metals and small metabolites away from extracellular and pathogen-accessible niches [21,34]. Importantly, this reduction does not necessarily reflect a loss of nutrients, but rather a reprogramming of their distribution, favoring storage within hepatocytes, immune cells, or intracellular compartments where microbial access is restricted [35]. At the cellular level, the acute-phase response reshapes subcellular nutrient landscapes, imposing compartment-specific limitations on invading pathogens. For example, through coordinated transporter activity within professional phagocytes, metals such as iron, zinc, and manganese are selectively excluded from phagosomes to starve phagocytosed pathogen while copper is intentionally transported into phagosomes to toxify the engulfed pathogen [21,36]. At the same time, cytosolic and mitochondrial pools are tightly regulated to support immune effector functions and redox balance [21,36]. Similarly, nutrient trafficking through the endosomal and secretory pathways is modified to limit microbial acquisition while maintaining host biosynthetic capacity [37]. Collectively, these processes underscore that nutritional immunity functions primarily through control of functional availability within defined compartments, rather than by reducing overall nutrient concentrations, thereby integrating immune defense with systemic and cellular metabolic homeostasis. This section presents the various strategies employed by the host to exert nutritional immunity.

### 2.1. Iron Limitation: A Key Mechanism of Nutritional Immunity

**Figure 2:**
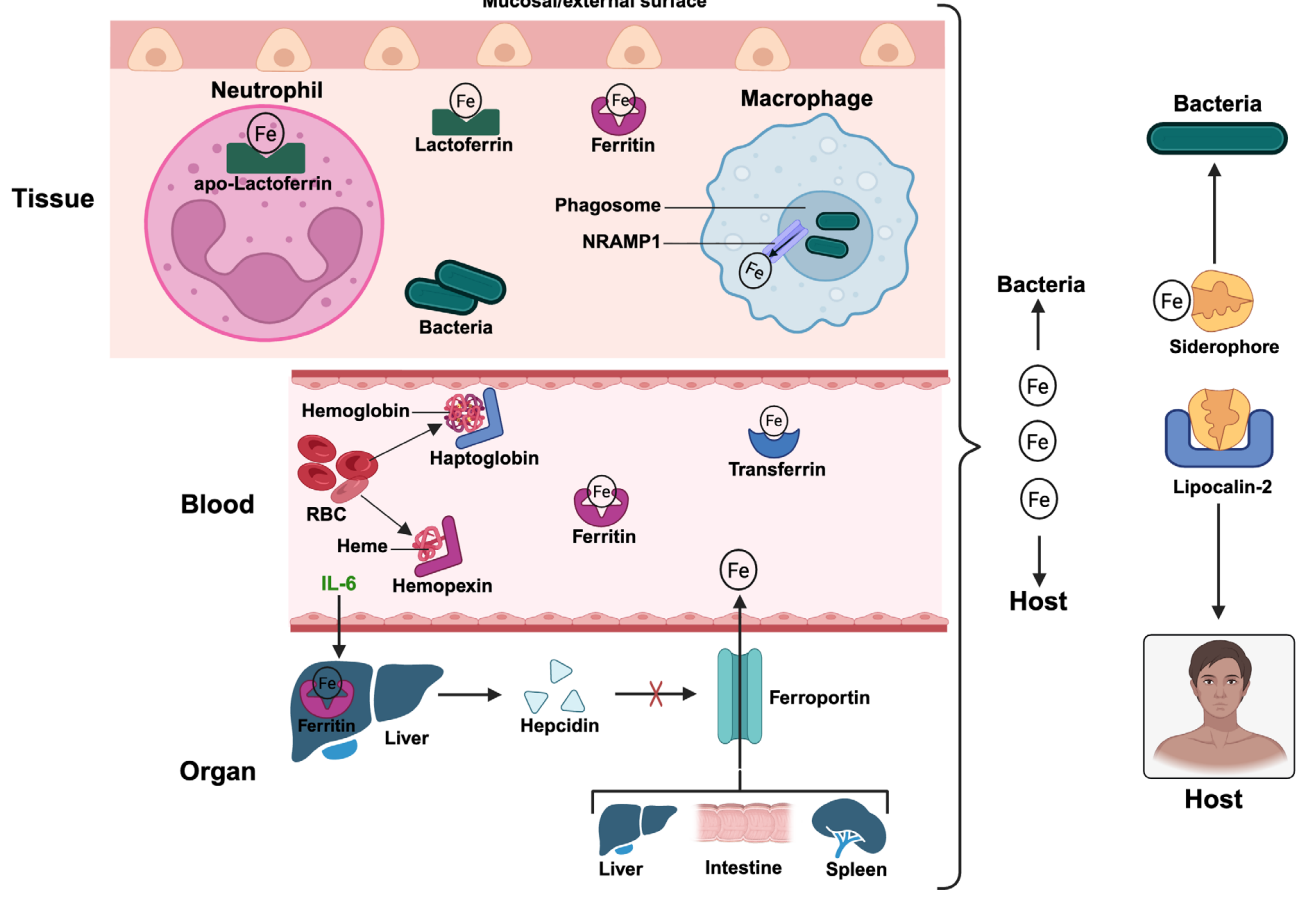
Iron sequestration by the host and microbial acquisition strategies. Upon sensing the pathogen, the host deploys mechanisms to hide iron (Fe) from the pathogen. For example, the liver is triggered by interleukin-6 (IL-6) to produce hepcidin which in turn degrades ferroportin, a major iron exporter. The degradation of ferroportin suppresses iron export into circulation, withholding iron in cells of the liver, intestine, and spleen. Other proteins deployed by host to sequester iron includes transferrin, which binds Fe in blood, limiting extracellular Fe; apo-lactoferrin, a high-affinity Fe-binding glycoprotein in mucosal secretions and neutrophil granules; ferritin, an intracellular Fe storage, that restricts cytoplasmic Fe availability; hemopexin, which binds free heme, limiting bacterial access to the Fe in heme; haptoglobin, which binds free hemoglobin (Hb), preventing bacterial Hb uptake; lipocalin-2, which binds bacterial siderophores, thereby, blocking Fe scavenging; and natural resistance-associated macrophage protein 1 (NRAMP1), which pumps Fe out of phagosomes to deprive intracellular bacteria. In response, the pathogen, also deploy counter mechanisms that allow them to still access these critical nutrients, including bacterial siderophores, which are secreted molecules that chelate Fe with very high affinity.

Iron is indispensable for almost all living organisms owing to its role in enzymatic reactions, oxygen transport, DNA synthesis, and cellular respiration (Table 1) [54]. Figure 2 illustrates the mechanisms deployed by the host to sequester iron from invading pathogens and how the pathogens respond to counter host limitation strategies. During infection, the host withholds iron using a pool of proteins, including ferritin, lactoferrin, transferrin, hemopexin, and haptoglobin, which bind iron and iron-containing complexes in extracellular and intracellular compartments [7]. For example, ferritin, lactoferrin, and transferrin binds free iron, hemopexin binds free heme, while haptoglobin binds free hemoglobin. It is important to note that the lactoferrin in neutrophil granules are predominantly in the iron-free (apo) form [40]. Collectively, these proteins directly and indirectly deprive invading pathogens access to iron and iron sources within the body. Additionally, the liver-derived peptide hormone hepcidin plays a pivotal role by degrading ferroportin, the primary iron exporter, thereby lowering serum iron levels and trapping iron in storage sites like macrophages, intestinal cells, splenocytes, and hepatocytes [11]. This strategy of iron sequestration is particularly effective against extracellular pathogens that depend on free iron for replication [55]. In phagocytes like macrophages, iron is actively hidden away from the phagosome and subsequently restricted from specialized pathogen-containing compartments to starve the invading pathogen. For example, following phagocytosis of an invading pathogen, macrophages actively remove ferroportin from the phagosomal membrane to prevent iron from being pumped into the phagosome lumen. Similarly, NRAMP1 actively transports iron out of the acidified phagosomal lumen into the macrophage’s cytoplasm [22,56]. The iron is instead transported back to the plasma membrane via membrane trafficking pathways involving SNARE proteins and the N-ethylmaleimide–sensitive factor (NSF) [57]. These sequestration efforts disrupt microbial enzymatic functions, especially those involved in DNA synthesis, respiration, and oxidative stress resistance, ultimately helping the host to repel the infection. However, many bacteria have developed countermeasures, including the production of siderophores, which are small, high-affinity iron-chelating molecules that scavenge iron from host proteins [58]. Pathogens have diversified their siderophore structures to evade host defenses such as lipocalin-2, which binds and neutralizes siderophores [59]. *Escherichia coli* and *Salmonella enterica*, for instance, synthesize covert siderophores like salmochelin, a glycosylated derivative of enterobactin that is not recognized by lipocalin-2 [60,61]. This structural modification permits pathogens to sustain iron acquisition under immune pressure. Diversification also includes the production of multiple siderophores with unique chemical properties. For example, *Pseudomonas aeruginosa* produces both pyochelin and pyoverdine, which differ in regulatory control and affinity, allowing the pathogen to adapt to varying iron availability [62]. Similarly, *Yersinia pestis* synthesizes yersiniabactin, which not only chelates iron but also binds copper, playing a twofold role in metal acquisition and detoxification [63]. Stealth siderophores are often encoded within pathogenicity islands and controlled by iron-responsive transcription factors such as Fur [64]. These systems are highly connected with virulence, as iron acquisition is critical for replication and immune evasion. Meanwhile, iron limitation also influences host immunity beyond direct antimicrobial effects. For instance, iron deprivation can modulate macrophage polarization, increase oxidative burst, and regulate cytokine production [65]. Moreover, iron status affects the outcome of infections such as tuberculosis and malaria, where both iron deficiency and overload can aggravate disease [66]. Iron regulation, therefore, is a double-edged sword that must be well balanced to optimize host defense. Notably, iron chelation has been therapeutically explored as an adjunct to antimicrobial therapy [67,68,69]. For example, agents like deferiprone and deferoxamine can diminish iron availability to pathogens, although they must be used with care to avert the impairment of host hematopoiesis and immune function [70]. A complete understanding of the nuances of iron metabolism during infection is necessary for developing safe and effective interventions that leverage nutritional immunity.

### 2.2. Zinc and Manganese: Essential Metals in Host–Pathogen Interactions

**Figure 3:**
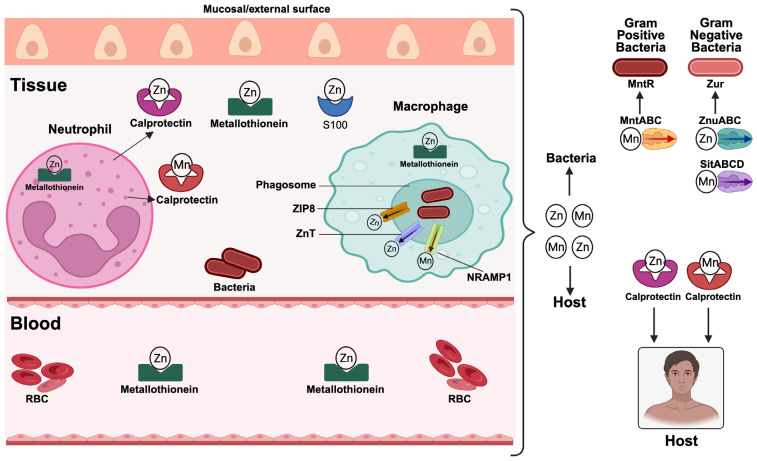
The zinc and manganese tug-of-war at the host–pathogen interface. The host deploys a number of strategies to actively sequester zinc (Zn) and manganese (Mn) from invading pathogens. These host strategies include calprotectin, a neutrophil protein that sequesters Zn and Mn; Zrt, Irt-like proteins 8 (ZIP8) and Zinc transporter (ZnT), which control intracellular Zn flux to deprive intracellular pathogens; natural resistance-associated macrophage protein 1 (NRAMP1), which pumps Mn out of phagosomes to deprive intracellular bacteria; and metallothionein, which bind zinc in blood circulation and inside immune cells, hiding zinc from pathogens. Gram-positive bacterial pathogens, in response, deploy high-affinity transporters like the MntABC transporter, a high-affinity Mn uptake system controlled by the metal repressor MntR. Gram-negative bacterial pathogens, in response, deploy high-affinity transporters like the ZnuABC system, a high-affinity Zn transporter regulated by the metal repressor Zur; and SitABCD, which helps pathogens acquire Mn intracellularly.

Zinc and manganese are critical cofactors for several microbial enzymes, including those involved in metabolic regulation, oxidative stress resistance, and DNA replication (Table 1) [9]. Thus, by reducing manganese availability, for example, the host leaves the pathogens prone to reactive oxygen species, facilitating microbial killing [65]. Figure 3 diagrammatically presents how the host attempts to restrict zinc and manganese from invading microbes as well as how the microbes respond to counter host withholding strategies. The host immune cells limit pathogen access to these metals through transporters and secreted molecules [5,71]. For example, calprotectin, a neutrophil-derived protein, chelates these metals at sites of infection and inflammation [9,72]. Notably, calprotectin (the S100A8/S100A9 heterodimer), majorly in the apo form, represents one of the most abundant antimicrobial proteins in innate immunity, making up approximately 40–60% of total cytosolic protein in human neutrophils [73,74]. Upon neutrophil activation, degranulation, or cell death, apo-calprotectin is released into the extracellular milieu, where it exerts potent nutritional immunity by chelating zinc and manganese with high affinity, thereby depriving extracellular and mucosal pathogens of essential metals [9,21,48]. In blood circulation and inside immune cells, metallothioneins bind zinc and hide them from pathogens [5]. Extracellularly, zinc is sequestered by zinc-chelating proteins known as the S100 proteins [75]. Phagocytes like macrophages actively keep manganese away from the phagosome and subsequently restrict this critical nutrient from specialized compartments harboring the pathogen to deprive the invading pathogen. For example, upon phagocytosis of an invading pathogen, macrophages actively deploy NRAMP1 and the ZIP-family transporter (ZIP8, ZnT) to transport manganese and zinc out of the acidified phagosomal lumen into the macrophage’s cytoplasm [22,56]. These sequestration efforts impair microbial enzymatic functions, especially those necessary for DNA replication and oxidative stress resistance, essentially helping the host fight off the infection. In response to this metal limitation imposed by the host, pathogens upregulate high-affinity transporters for zinc and manganese. For example, the ZnuABC system in Gram-negative bacteria and the MntABC system in Gram-positive bacteria promote zinc and manganese uptake and are critical for survival under calprotectin-mediated sequestration [21]. These transporters are often regulated by metal-responsive repressors such as Zur [76,77] and MntR [78,79], which fine-tune expression based on intracellular metal levels. *Staphylococcus aureus* exemplifies this strategy by expressing multiple metal transporters and metalloregulatory proteins [80]. It uses the MntABC system for manganese uptake and adapts its metalloproteome to replace scarce metals with available alternatives [81]. This flexibility permits pathogens to sustain enzymatic function and combat oxidative stress even under metal deprivation. Transporter upregulation is usually integrated with virulence gene expression. For example, *Salmonella enterica* activates SitABCD [82] and ZnuABC [83] during intracellular infection, enhancing survival in macrophage phagosomes. These systems facilitate nutrient acquisition and contribute to immune evasion and persistence, underscoring their dual role in pathogenesis. Furthermore, zinc limitation also affects host immunity as it is critical for cytokine production, T cell development, and barrier integrity [84]. For instance, at the cellular level, zinc acts as a structural and catalytic cofactor for hundreds of enzymes and transcription factors, including those involved in cytokine gene expression and signal transduction pathways such as JAK–STAT and NF-κB [85]. Also, zinc availability is particularly critical for T lymphocyte development and function, influencing thymic maturation, T cell receptor signaling, and the balance between pro-inflammatory Th1/Th17 responses and regulatory T cells [86]. In addition to shaping adaptive immunity, zinc is indispensable for maintaining epithelial barrier integrity and innate immune defenses. For example, zinc deficiency compromises tight junction stability in epithelial tissues, increasing susceptibility to pathogen invasion at mucosal surfaces [87]. During infection, zinc redistribution promotes the activation of immune cells while restricting microbial access [88]. However, excessive zinc sequestration can compromise host function [89], which underlines the need for careful regulation of zinc homeostasis. This twofold role of zinc and manganese in microbial metabolism and immune defense underpins their relevance in nutritional immunity.

### 2.3. Copper and Zinc Toxicity and Pathogen Detoxification Strategies

**Figure 4:**
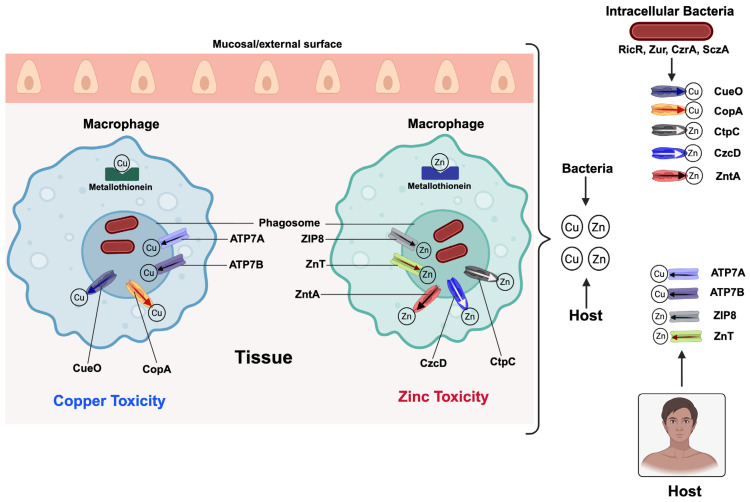
Host-mediated copper and zinc intoxication of pathogens and counterstrategies. Upon phagocytosis of an invading pathogen, macrophages send in excess amounts of copper and zinc into phagosomes and pathogen-containing compartments using special transporters like ATP7 transporters (ATP7A, ATP7B), which transport copper into the phagosome; zinc transporters (ZIP8, ZnT), which send in zinc into the phagosome. To reduce the toxic levels of these metals within their containing compartments, pathogens employ several strategies, including copper efflux pumps like CopA and CueO; high-affinity zinc efflux systems like P-type ATPases (ZntA, CtpC) and cation diffusion facilitator (CDF) transporters (CzcD). These efflux systems regulated by transcriptional factors like RicR, Zur, CzrA, and SczA, actively export excess zinc and copper out of the phagosome. During this exchange, metallothionein binds copper as needed within macrophage to buffer intracellular levels and to avert collateral damage.

Contrary to iron and manganese, the host can weaponize copper and zinc as toxic antimicrobial agents. Interestingly, in the case of zinc, the host can use either its restriction or intoxication to attack invading pathogens. Figure 4 illustrates how the host deploys copper and zinc to intoxify invading pathogens and how the pathogens respond to counter these host-mediated toxicity efforts. During intracellular infections, macrophages accumulate copper and zinc in phagosomes via ATP7 transporters (ATP7A, ATP7B) and zinc transporters (ZIP8, ZnT family), respectively [43,90]. These toxic metal levels overwhelm microbial detoxification systems and interfere with critical metabolic processes. For instance, the correct metalation of bacterial enzyme cofactors is disrupted when excess copper and zinc replace iron and manganese [91]. Excess metals in phagosomes catalyze the generation of reactive oxygen species and impair microbial iron–sulfur clusters [92]. Additionally, copper and zinc toxicity compromise pathogen’s DNA repair, respiration and redox balance [93]. Pathogens respond by expressing chaperones, copper efflux pumps like CopA and CueO, as well as detoxifying enzymes that alleviate copper-induced damage [94]. *Mycobacterium tuberculosis*, for example, activates the RicR regulon to handle copper stress during intracellular infection [95]. Intracellular pathogens counter zinc stress predominantly by inducing high-affinity zinc efflux systems, including P-type ATPases (such as ZntA and CtpC) and cation diffusion facilitator (CDF) transporters (such as CzcD). These efflux systems actively export excess zinc away from the phagosome and pathogen-containing compartments and are therefore critical for survival within macrophages [41,96]. Concomitantly, pathogens transcriptionally reprogram metal homeostasis pathways via zinc-responsive regulators, including Zur, CzrA, or SczA, leading to repression of zinc uptake systems and coordinated activation of detoxification and efflux genes [21,91]. Together, these strategies enable intracellular pathogens to neutralize host-imposed zinc toxicity and maintain metal homeostasis within the otherwise hostile phagosomal niche. Striking a balance between antimicrobial efficacy and host toxicity is an important consideration in copper and zinc-based immunity. For instance, host copper homeostasis is highly regulated via the use of metallothioneins and copper-binding proteins to buffer intracellular levels and avert collateral damage [97]. Therapeutically, copper-enhancing agents and copper-mimetic compounds [98,99], as well as zinc oxide nanoparticles [100,101,102], are being explored for their antimicrobial potentials. These strategies aim to exploit pathogen vulnerabilities to copper and zinc stress while maintaining host homeostasis. Further research into copper and zinc trafficking and regulation during infection may produce novel approaches to utilizing these potent antimicrobial metals.

### 2.4. Magnesium Limitation and Membrane Integrity

Magnesium is necessary for stabilizing cellular membranes, nucleic acids, and ribosomes in both host and microbes [103]. During infection, the host restricts magnesium availability within phagosomes, disrupting bacterial membrane integrity and signaling pathways [104]. This strategy is precisely potent against intracellular pathogens that depend on magnesium for survival in acidic vacuolar environments. *Salmonella enterica* senses magnesium limitation through the PhoP/PhoQ two-component system, which activates genes that reinforce resistance to acid stress and antimicrobial peptides [105]. This system also controls virulence factors, connecting magnesium sensing to pathogenicity. *S. enterica* mutants deficient in PhoP/PhoQ signaling exhibit reduced survival in macrophages, highlighting the significance of magnesium adaptation [106]. Magnesium starvation from pathogens makes them vulnerable to host defense mechanisms [4]. It impairs ATP synthesis, disrupts membrane potential, and heightens susceptibility to antibiotics and immune effectors. The host takes advantage of these vulnerabilities by modulating magnesium transport and buffering systems during infection [4]. Despite its significance, magnesium restriction is not as well-characterized as iron or zinc limitation. Future research should probe the molecular mechanisms of magnesium sequestration, its impact on immune cell function, and its potential as a therapeutic target. A deeper understanding of magnesium dynamics may present new strategies for controlling intracellular infections.

### 2.5. Sulfur and Nitrogen Limitation and Pathogen Adaptation

Sulfur and nitrogen are vital elements for microbial survival, which serve as building blocks for nucleotides, amino acids, and cofactors [107,108]. The host can limit pathogen access to these nutrients through enzymatic degradation and metabolic rerouting. For nitrogen, a major host strategy is the depletion of arginine [109] and tryptophan [110]. Arginine is consumed by host arginase, which diminishes its availability for the synthesis of nitric oxide and microbial growth, while tryptophan is broken down by indoleamine 2,3-dioxygenase (IDO), generating immunomodulatory metabolites like kynurenine [110]. Interestingly, pathogens have also developed mechanisms to bypass these restrictions. For example, *Chlamydia trachomatis* encodes tryptophan synthase, with which it synthesizes tryptophan de novo when host levels drop [111]. Similarly, *Mycobacterium tuberculosis* upregulates nitrogen absorption pathways under nutrient stress, promoting the bacteria’s survival within macrophages [112]. These adaptations point to the metabolic flexibility of intracellular pathogens in response to nitrogen limitations orchestrated by the host. Sulfur metabolism is not much characterized in nutritional immunity, but it is gaining attention. Meanwhile, amino acids that contain sulfur, such as methionine and cysteine, are necessary for microbial antioxidant defenses and protein synthesis [113]. The host may decrease sulfur availability by downregulating the expression of transporters and enzymes involved in sulfur intake [107]. Additionally, oxidative stress caused by immune cells can oxidize sulfur compounds, making them inaccessible to the invading pathogens [114]. An in-depth understanding of how the host restricts sulfur and nitrogen will unveil new avenues for antimicrobial development. For example, targeting microbial sulfur assimilation or exploiting nitrogen starvation responses could facilitate pathogen clearance. Moreover, modulating host enzymes like arginase and IDO offers potential for immunotherapy, particularly in chronic pathogenic infections and cancer, where nutrient metabolism is intertwined with immune regulation.

### 2.6. Vitamin Sequestration and Pathogen Adaptation

**Figure 5:**
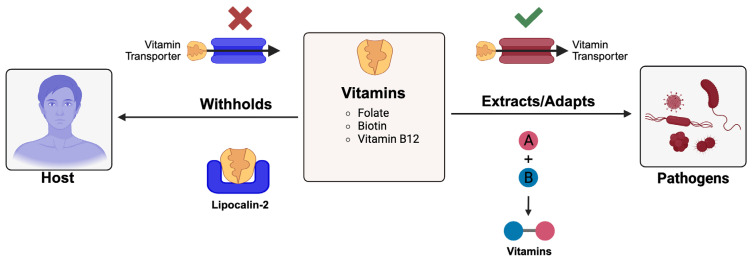
Vitamin sequestration and pathogen counterstrategies. The host restricts vitamins such as folate, biotin, and vitamin B12, from invading pathogens by using proteins like lipocalin-2 to withhold vitamins, downregulating vitamin transporters, and downregulating vitamin receptors. Some pathogens bypass this hurdle by synthesizing vitamins from the scratch as well as upregulating and deploying high-affinity vitamin transporters to acquire vitamins from the host.

Vitamins are critically required by both host and pathogens as cofactors in metabolic pathways and enzymatic reactions (Table 1) [115,116]. Figure 5 shows host strategies for depriving pathogens of vitamins and pathogen counterstrategies. Following bacterial infection, the host can restrict access to vitamins such as folate, biotin, and vitamin B12, which creates an impediment for microbial metabolism [117]. This strategy is particularly potent against microbes that depend on host-derived vitamins due to incomplete biosynthetic pathways. Lipocalin-2, classically known for binding bacterial siderophores, has also been implicated in withholding vitamins. It can bind vitamin-like molecules and interfere with microbial uptake, extending its role beyond iron restriction [118]. Additionally, host cells may downregulate vitamin transporters or alter vitamin metabolism during infection, further limiting pathogen access. For example, during human cytomegalovirus (CVM) infection, host cells have been shown to downregulate vitamin D receptor (VDR), impacting the vitamin D system and influencing immunity [119]. Some pathogens overcome this roadblock by synthesizing vitamins de novo. For example, *Salmonella typhimurium* possesses a full vitamin B12 biosynthesis pathway, which permits it to flourish in the nutrient-limited gastric mucosa [120]. Also, *Mycobacterium tuberculosis* expresses high-affinity transporters for biotin and folate, which are critical for fatty acid synthesis and nucleotide metabolism, respectively [121]. Fungal pathogens like *Candida albicans* and *Cryptococcus neoformans* also possess redundant vitamin acquisition systems, enabling survival in nutrient-limited niches [122,123]. Therapeutically, vitamin restriction presents a novel angle for antimicrobial intervention as inhibitors of microbial vitamin biosynthesis or transport could selectively hamper pathogen metabolism without affecting host cells. Overall, vitamin sequestration is a budding edge in nutritional immunity, and understanding how pathogens obtain and utilize these micronutrients could uncover new weak points for therapeutic targeting.

### 2.7. Glucose Restriction and Pathogen Adaptation

Glucose, a major carbon source, is fundamental to microbial biosynthetic processes and energy production (Table 1) [124,125]. The host restricts carbon availability for the invading pathogens by modifying transporter expression, nutrient compartmentalization, and metabolic flux [126,127]. Upon activation, immune cells utilize large amounts of glucose and glutamine, denying pathogens of these substrates and creating a competitive metabolic environment [25]. Pathogens adapt by resorting to alternative carbon sources or scavenging metabolites generated by the host [128]. This metabolic adaptability is important for survival in nutrient-restricted environments such as inflamed tissues or intracellular compartments. For instance, *Listeria monocytogenes* circumvents glucose limitation during intracellular growth by transitioning to host-derived acetate and glycerol [129,130]. This switch is governed by PrfA, a master virulence regulator that integrates metabolic and pathogenic signals [129]. Similarly, *Mycobacterium tuberculosis* displays extreme metabolic plasticity by utilizing host lipids as carbon sources during intracellular infection. For example, it upregulates genes involved in β-oxidation and the glyoxylate shunt, allowing it to persist in macrophages where glucose is scarce [131]. This adaptation not only supports energy production but also manipulates immune responses by reshaping host lipid metabolism. Also, *Salmonella enterica* expresses several carbon transporters and metabolic enzymes that help it to thrive in nutrient-poor vacuoles [112]. These adaptations demonstrate the metabolic versatility required for intracellular survival. Carbon source flexibility often supports virulence and immune evasion, in that pathogens that can exploit host metabolites are better prepared to survive nutrient limitation and immune activation. Therefore, metabolic rewiring represents a strategic adaptation that improves microbial fitness and pathogenicity in diverse host environments. Glucose and carbon source restriction also shapes immune responses. For example, metabolites such as lactate and succinate, generated during glycolysis and the TCA cycle, modulate cytokine production and immune cell differentiation [132]. Therefore, nutrient competition between host and pathogen not only affects microbial survival but also influences the immune landscape. Consequently, targeting microbial carbon metabolism offers therapeutic potential. For example, inhibitors of key glycolytic enzymes, glycerol uptake, or acetate metabolism could hinder pathogen replication. Moreover, modulating host metabolism to increase nutrient competition or immune activation may offer synergistic benefits in infection control.

### 2.8. Amino Acid Deprivation and Metabolic Reprogramming

Amino acids are necessary for protein synthesis, metabolic regulation, and immune signaling (Table 1) [133,134]. The host limits amino acid availability to pathogens through enzymatic degradation and transporter modulation. For instance, tryptophan is broken down by IDO, while arginine is used up by arginase, reducing their availability to pathogens and modulating immune responses [135,136]. These mechanisms are particularly efficacious against intracellular pathogens that depend on host amino acids [46]. In response, pathogens either upregulate biosynthetic pathways or scavenge amino acids from the host. For example, *Chlamydia trachomatis* encodes tryptophan synthase to generate tryptophan *de novo*, counteracting host IDO-mediated tryptophan depletion, and enabling sustained replication in epithelial cells [111]. This enzyme is controlled by nutrient availability and immune signals, highlighting its role in adaptation. Similarly, *Mycobacterium tuberculosis* [137] and *Salmonella enterica* [138] also express amino acid biosynthetic enzymes and transporters that are upregulated during intracellular infection. These systems permit the pathogens to thrive in vacuoles where amino acid levels are tightly controlled [112]. Amino acid scavenging is often coupled with stress responses and the expression of virulence genes, which altogether improve survival under immune pressure. Further, some pathogens manipulate host autophagy to access amino acids. *Toxoplasma gondii* [139] and *Coxiella burnetii* [140], for instance, both induce host autophagy to release nutrients into the parasitophorous vacuole. This strategy not only supplies amino acids to the pathogens but also modulates host immunity, illustrating the multidimensional role of amino acid acquisition in pathogenesis. Further, amino acid deprivation also reprograms host immunity by activating stress responses and metabolic checkpoints that facilitate pathogen clearance. For instance, glutamine and leucine availability influence mTOR signaling, which regulates T cell activation and differentiation [141]. Thus, amino acid metabolism is a junction of nutritional immunity and immune regulation. Manipulating amino acid availability presents therapeutic opportunities for infection control and immunomodulation. Arginase modulators, IDO inhibitors, and amino acid analogs are being researched in infectious disease and cancer. Understanding the context-specific effects of amino acid deprivation will be key to safely and effectively exploiting these strategies.

### 2.9. Nutritional Immunity with Lipids: Nutrients and Niche Modulators

Beyond metals and small metabolites, host lipids, including fatty acids, cholesterol, phospholipids, and lipid droplets (LDs), are both critical nutrient sources and structural building blocks for many pathogens, and they actively shape intracellular niches (Table 1) [142]. Host control over lipid trafficking and storage therefore functions as a form of nutritional immunity that can limit pathogen growth or, paradoxically, be subverted to enhance pathogen persistence [37,143]. Infected cells reprogram lipid metabolism. For example, macrophages and other innate cells ramp up the uptake and esterification of cholesterol, synthesize neutral lipids, form LDs, and alter fatty-acid flux. LDs act as metabolic hubs and may be “weaponized” by the host, for example, by concentrating antimicrobial lipids or lipid-derived mediators, or, if hijacked, provide a nutrient depot for invaders [143,144,145]. Intracellular bacteria and parasites exploit host lipids in multiple ways. These include import and catabolism of host cholesterol as seen in *Mycobacterium tuberculosis* [131], redirection of host fatty-acid metabolism [144], and interception of LDs and secretory trafficking to obtain membranes and energy as observed in *Chlamydia*, *Salmonella*, and *Toxoplasma* [142]. Dedicated bacterial systems such as *M. tuberculosis* cholesterol uptake and degradation pathways, coordinated by proteins like LucA, enable utilization of host sterols for persistence [131,142,146]. Targeting lipid access or utilization with statins, inhibitors of microbial cholesterol catabolism, modulation of LD biology, or host-directed metabolic reprogramming, offers promising adjunctive strategies to traditional antimicrobials especially in the wake of rising antibiotic resistance. However, given that lipids are central to host physiology, therapeutic windows are narrow and require precision [37,144].

### 2.10. Direct Antimicrobial Actions

Nutritional immunity also involves direct antimicrobial actions mediated by nutrient-binding proteins and metabolic byproducts. For example, calprotectin not only chelates zinc and manganese but also punctures microbial membranes and blocks enzymatic activity, exerting bacteriostatic effects [147]. Also, lipocalin-2 binds bacterial siderophores, hampering iron uptake and triggering oxidative stress in pathogens [50]. These proteins act as both nutrient scavengers and antimicrobial effectors. Oxidative radicals, such as reactive oxygen species (ROS) and nitrogen oxygen species (RNS), generated during immune activation, further facilitate nutritional immunity. For instance, these molecules oxidize and degrade microbial cofactors, such as iron–sulfur clusters and thiol groups, compromising metabolic function and replication [148]. Also, copper and zinc, concentrated in phagosomes, catalyze ROS production, which impairs microbial redox balance, enhancing killing efficiency [94]. Metabolic byproducts also play a role in enhancing nutritional immunity. As an example, kynurenine, produced from tryptophan catabolism via IDO, has immunomodulatory and antimicrobial properties. It suppresses T cell proliferation and triggers apoptosis in infected cells, facilitating pathogen clearance [135]. Similarly, lactate and succinate function as signaling molecules that shape immune cell behavior during infection. Lactate, generated through aerobic glycolysis in activated immune cells, accumulates in inflamed tissues and can modulate immune responses in a context-dependent manner. For instance, elevated lactate concentrations suppress excessive inflammation by inhibiting histone deacetylases and dampening NF-κB–dependent cytokine production, thereby preventing immunopathology [149,150]. At the same time, lactate alters the local microenvironment in ways that restrict pathogen proliferation, as many bacteria and parasites are sensitive to acidic conditions and metabolic stress induced by lactate accumulation [151]. Succinate, a tricarboxylic acid (TCA) cycle intermediate, similarly acts as a key immunometabolic signal during infection. For example, succinate accumulates in macrophages following inflammatory stimulation and stabilizes hypoxia-inducible factor-1α (HIF-1α), thereby promoting interleukin-1β (IL-1β) production and antimicrobial effector functions [132]. However, extracellular succinate can also signal through the succinate receptor SUCNR1, influencing dendritic cell maturation and modulating adaptive immune responses [152]. Overall, these direct antimicrobial actions complement other nutritional immunity strategies, creating an unwelcome environment for pathogens. Essentially, we see the integration of metabolic, immunological, and biochemical defenses in nutritional immunity. Future research should explore the therapeutic potential of these antimicrobial molecules, including their use as adjuvants, antimicrobial agents, or immunomodulators in treating infectious diseases.

## 3. Nutritional Immunity in the Context of Pathogen Classes and Niches

Nutritional immunity is not a monolithic defense strategy. Rather, it is dynamic, pathogen-specific, and shaped by the ecological niche, virulence mechanisms, and metabolic dependencies of invading microbes. This section examines how different categories of pathogens across infection niches, including extracellular, intracellular, eukaryotic, and viruses, interact with host nutrient sequestration systems, and how comparative genomics illustrate evolutionary adaptations in nutrient acquisition.

### 3.1. Extracellular Pathogens: Confronting Nutrient Sequestration Head-On

Extracellular pathogens, which dwell outside host cells, are directly exposed to the host’s nutrient-sequestering proteins at mucosal surfaces, tissues, and the bloodstream [5]. For example, *Neisseria meningitidis* must compete with iron-binding proteins like lactoferrin and transferrin in the cerebrospinal fluid and blood [153]. To overcome this, *N. meningitidis* expresses transferrin-binding proteins like TbpA and TbpB, which directly extract iron from host transferrin [154,155]. This direct contest for iron is a major attribute of extracellular pathogens, which often develop surface receptors that mimic host ligands. A different strategy is employed by *Streptococcus pneumoniae*, which expresses pneumococcal surface protein A (PspA) that suppresses complement deposition and indirectly protects against nutritional immunity by inhibiting immune activation [156,157]. *S. pneumoniae* also uses ABC transporters to obtain zinc and manganese, opposing sequestrations mediated by calprotectin [158]. These metal acquisition systems are highly regulated and often connected with virulence gene expression, highlighting the twofold role of nutrient sensing in pathogenesis. *Staphylococcus aureus* and *Pseudomonas aeruginosa* are particularly proficient at surviving in nutrient-constrained settings. For example, *S. aureus* expresses iron-regulated surface determinant (Isd) proteins, which extract heme from hemoglobin and deliver it across the bacterial envelope [159]. Similarly, *P. aeruginosa* synthesizes multiple siderophores, including pyochelin and pyoverdine, which scavenge iron from host proteins and are regulated by quorum sensing [62,160]. These systems are critical for survival in iron-deprived niches such as abscesses. Another extracellular pathogen, *Escherichia coli*, especially uropathogenic strains (UPEC), deploys covert siderophores like salmochelin to elude lipocalin-2, a host protein that binds and neutralizes bacterial siderophores [60,161]. Multiple iron acquisition systems are also expressed by UPEC, allowing it to adjust to different iron sources in the urinary tract [162]. Altogether, extracellular pathogens express a diverse array of nutrient acquisition strategies that demonstrate their exposure to host defenses and their need for rapid adaptation.

### 3.2. Intracellular Pathogens: Navigating the Nutrient Desert Within

Intracellular pathogens contend with a different challenge of surviving within host cells, where nutrients are highly regulated, often withheld in organelles, and actively expelled from pathogen-containing compartments [163]. Interestingly, this group of pathogens has also evolved various strategies for overcoming these nutrient limitations. *Mycobacterium tuberculosis*, for example, resides within macrophage phagosomes [164], where iron and zinc are restricted [165]. To overcome this limitation, the pathogen expresses siderophores called mycobactins and amplifies the expression of iron acquisition genes in low-iron situations [166]. Additionally, *M. tuberculosis* modulates host lipid metabolism to acquire fatty acids as carbon sources, revealing its metabolic flexibility [131]. *Salmonella enterica* survives within *Salmonella*-containing vacuoles (SCVs) in macrophages and epithelial cells [167]. It recognizes magnesium limitation through the PhoP/PhoQ system and activates genes that increase survival in magnesium-limited environments [168]. It also expresses ZnuABC and SitABCD transporters to acquire zinc and manganese, respectively, countering calprotectin-mediated sequestration [169,170]. These adaptations are necessary for the pathogen’s intracellular replication and systemic dissemination. *Toxoplasma gondii*, an obligate intracellular parasite, lives in a parasitophorous vacuole that is largely detached from host endocytic pathways [171]. It scavenges host amino acids and cholesterol through specialized transporters and manipulates host autophagy to access nutrients [172,173]. Nutrient limitation triggers stage conversion from tachyzoite to bradyzoite, a dormant form that enhances persistence [174]. This developmental plasticity is a trademark of intracellular eukaryotic pathogens. *Coxiella burnetii*, *Chlamydia trachomatis*, and *Rickettsia prowazekii* each occupy distinct intracellular niches [46]. For example, *C. burnetii* thrives in acidic lysosome-like vacuoles and expresses transporters for amino acids and metals [175]. *C. trachomatis* resides in an inclusion body and encodes tryptophan synthase to compensate for host IDO-mediated tryptophan depletion [111]. *R. prowazekii*, which replicates in the cytosol, scavenges host amino acids and ATP, reflecting its reductive genome and reliance on host metabolism [46]. Aside from acquiring nutrients, intracellular pathogens actively modulate host processes to evade nutritional immunity. For example, *Leishmania* spp. alter phagosome maturation and acidification, creating a niche for optimal nutrient acquisition and minimized immune detection [176]. These modifications are facilitated by surface glycoconjugates and secreted effectors that interfere with host signaling. Similarly, *Mycobacterium tuberculosis* produces ESAT-6 and other effectors that block phagosome-lysosome fusion and suppress pro-inflammatory signaling [177]. These tactics not only keep the pathogen from immune clearance but also preserve access to intracellular nutrients, reinforcing the connection between immune evasion and nutritional adaptation. Overall, these pathogens embody diverse strategies used to navigate and survive intracellular nutrient deserts.

### 3.3. Eukaryotic Pathogens: Complexity, Redundancy, and Immune Evasion

Eukaryotic pathogens, including fungi, parasites, and protozoa, have complex life cycles and redundant metabolic pathways that help them evade nutritional immunity [4,6]. For example, *Plasmodium falciparum*, the protozoan causative agent of malaria, invades red blood cells and breaks down hemoglobin to access amino acids and heme [178,179]. It detoxifies heme into hemozoin and expresses transporters for iron and other metals [179]. Host iron status determines malaria severity, and iron supplementation can worsen infection, underscoring the delicate balance of iron homeostasis [16]. *Leishmania* species reside in macrophage phagolysosomes and express ZIP family transporters for iron and zinc [180]. They also synthesize surface glycoconjugates like lipophosphoglycan (LPG) that manipulate host immune responses, enhancing nutrient acquisition [181]. The ability of *Leishmania* to survive in acidic, nutrient-limited compartments highlights its evolutionary adaptation to unfriendly intracellular environments. Fungal pathogens such as *Candida albicans*, *Cryptococcus neoformans*, and *Aspergillus fumigatus* possess robust nutrient acquisition systems. For instance, *C. albicans* expresses siderophore transporters and ferric reductases to acquire iron, and its metabolic flexibility permits it to flourish in diverse host niches [182]. *C. neoformans* synthesizes melanin and capsule components that defend the pathogen against oxidative stress and facilitate iron uptake [183]. *A. fumigatus* expresses high-affinity iron transporters and secretes siderophores, which are critical for virulence in immunocompromised hosts [184]. These eukaryotic pathogens also dodge nutritional immunity through immune modulation, antigenic variation, and metabolic redundancy [185,186]. For example, *Plasmodium falciparum* reprograms red blood cells to enhance nutrient uptake and waste disposal [187]. It sends out proteins that form new permeability pathways, creating access to amino acids and glucose [188]. These alterations also decrease immune recognition, improving parasite survival and replication. Immune evasion strategies often involve modulation of host cytokine responses and antigen presentation [189]. Overall, the fact that their genomes encode multiple isoforms of nutrient transporters and enzymes coupled with their ability to modulate host cell signaling and immune responses adds a layer of complexity to their nutrient acquisition strategies, making them formidable adversaries in the context of nutritional immunity.

### 3.4. Nutritional Immunity in the Context of Viral Infections

Nutritional immunity has been predominantly studied in bacterial and fungal pathogenesis. Yet, accumulating evidence illuminates its relevance during viral infections. Unlike bacteria, viruses do not directly acquire metals for replication but rely heavily on host cellular metabolic processes that are metal-dependent, including enzymatic activity, DNA/RNA synthesis, and immune signaling [190]. Therefore, the host–virus interaction with nutritional immunity is largely indirect, mediated through regulation of metal homeostasis, nutrients redistribution, and modulation of host immune responses. Iron metabolism is strongly implicated in viral infections. Many viruses, including Human Immunodeficiency Virus (HIV), hepatitis C virus (HCV), and cytomegalovirus (CMV), exploit host iron-dependent pathways for replication [66]. High iron levels can enhance viral replication, while iron limitation via hepcidin upregulation or therapeutic chelation can suppress viral spread [191]. Conversely, iron overload in conditions such as hemochromatosis predisposes patients to more severe viral infections [192]. Zinc plays a twofold role in antiviral defense and viral pathogenesis. For instance, intracellular zinc bolsters antiviral immunity by promoting interferon signaling and stabilizing antiviral proteins, while zinc-finger antiviral protein (ZAP) directly restricts viral RNA [193]. Supplementation of zinc has been shown to lower the replication of coronaviruses, influenza virus, and HIV in vitro [193,194]. However, pathogens may oppose host zinc redistribution strategies. For instance, HIV can alter zinc transporter expression to favor its persistence in macrophages [193]. Copper and manganese also intersect with viral infections. For example, copper possesses intrinsic antiviral activity, disrupting viral proteins and nucleic acids, a property exploited in both innate immunity and copper-based surface coatings for infection control [195]. Meanwhile, manganese, is critical for the cGAS–STING pathway, a pivotal antiviral sensing mechanism, and its depletion impairs interferon-mediated viral clearance [196]. Taken together, nutritional immunity in viral infections represents a subtler and more host-centric phenomenon than in bacterial infections, where the pathogen directly competes for metals. Viruses rewire host nutrient availability to their benefit, while the host leverages nutrient redistribution to limit viral replication and enhance immune defense. This emerging field opens new therapeutic avenues, including modulation of iron homeostasis, zinc supplementation, and copper-based antivirals which are already under investigation in both preclinical and clinical settings [197,198,199,200,201].

### 3.5. Comparative Genomics: Mapping the Evolutionary Landscape of Nutrient Acquisition

Comparative genomics has elucidated the evolutionary trajectories of nutrient acquisition systems across diverse pathogens. By evaluating genome content, gene expression, and regulatory networks, researchers have spotted conserved and lineage-specific adaptations that depict ecological niches and host interactions. Pathogens often expand gene families involved in acquiring nutrients to increase redundancy and adaptability [202]. For instance, genes involved in siderophore biosynthesis and transport are frequently duplicated and organized into operons, enabling coordinated expression [203,204,205]. *Pseudomonas aeruginosa* has several operons for pyoverdine synthesis, each controlled by the availability of iron and environmental cues [206]. Operon architecture promotes rapid response to nutrient limitation, since genes encoding transporters, regulators, and biosynthetic enzymes are co-transcribed, ensuring efficient resource allocation. This organization is particularly visible in metal acquisition systems such as ZnuABC and SitABCD, which are tightly regulated by metal-responsive repressors [207]. The expansion of genes also supports functional diversification. The duplicated genes may develop new substrate specificities or regulatory mechanisms, elevating pathogen fitness in diverse environments [208]. This evolutionary strategy illustrates the selective pressure imposed by nutritional immunity and the need for pathogens’ metabolic versatility. Interestingly, intracellular pathogens often exhibit genome reduction, shedding redundant metabolic pathways while preserving or expanding nutrient transporters [46]. *Chlamydia* and *Rickettsia* species, for example, have streamlined genomes but encode specialized systems for extracting host metabolites like amino acids, nucleotides, and ATP, which illustrates their obligate intracellular lifestyle [209,210,211]. Reductive evolution is fueled by genome decay and host dependency. Genes that are no longer needed for extracellular survival are lost, while those involved in host interaction and nutrient uptake are preserved or expanded. While reductive evolution increases efficiency by transferring biosynthetic functions to the host, it simultaneously creates nutritional fragility for the pathogen. For example, the reliance of *Chlamydia* on host tryptophan makes it highly susceptible to IDO-mediated starvation [111]. Likewise, *Coxiella*’s requirement for host-derived lipids and amino acids restricts its replication to the specialized parasitophorous vacuole [212]. Contrastingly, facultative intracellular pathogens like *Salmonella* maintain versatile metabolic networks, allowing survival in both intracellular and extracellular environments [213]. Eukaryotic pathogens show extensive gene duplication and diversification in nutrient acquisition genes [214,215]. For example, *Plasmodium* species have several copies of enzymes and transporters involved in amino acid and metal metabolism, demonstrating their complex life cycle and host transitions [216]. Also, fungal pathogens possess expanded gene families for oxidative stress resistance and iron uptake, which corresponds with their capacity to colonize diverse tissues [217]. Comparative genomics also reveals horizontal gene transfer (HGT) which plays a critical role in the dissemination of nutrient acquisition and catabolism systems [218]. Pathogenicity islands commonly contain siderophore biosynthesis genes, metal transporters, and vitamin synthesis pathways obtained from other microbes [219]. *Yersinia pestis*, for example, acquired the yersiniabactin cluster via HGT, improving its iron acquisition capabilities [220]. Convergent evolution also determines nutrient acquisition strategies. Thus, unrelated pathogens may develop similar mechanisms to conquer host defenses, such as stealth siderophores or high-affinity transporters. This convergence reflects general selective pressures and underscores the functional importance of these adaptations [221]. HGT and convergence contribute to the speedy evolution of virulence characteristics. By acquiring and optimizing nutrient acquisition genes, pathogens can adapt to new hosts and niches, expanding their ecological range and pathogenic potential. These genomic insights reflects the evolutionary arms race between host nutritional immunity and microbial adaptation and have important translational implications. Pathogen-specific nutrient acquisition systems often display limited homology to host proteins, making them attractive targets for antimicrobial drug development and vaccine design [222]. For example, siderophore receptors, metal transport ATPases, and vitamin uptake systems have been explored as vaccine antigens or as entry points for “Trojan horse” antibiotics [221]. Thus, understanding the evolutionary dynamics of nutritional immunity not only deepens our knowledge of host–pathogen interactions but also reveals actionable vulnerabilities that can be exploited therapeutically.

## 4. Immunological Mechanisms and Regulation of Nutritional Immunity

### 4.1. Immune Sensing and Activation of Repressive Immunometabolism

**Figure 6:**
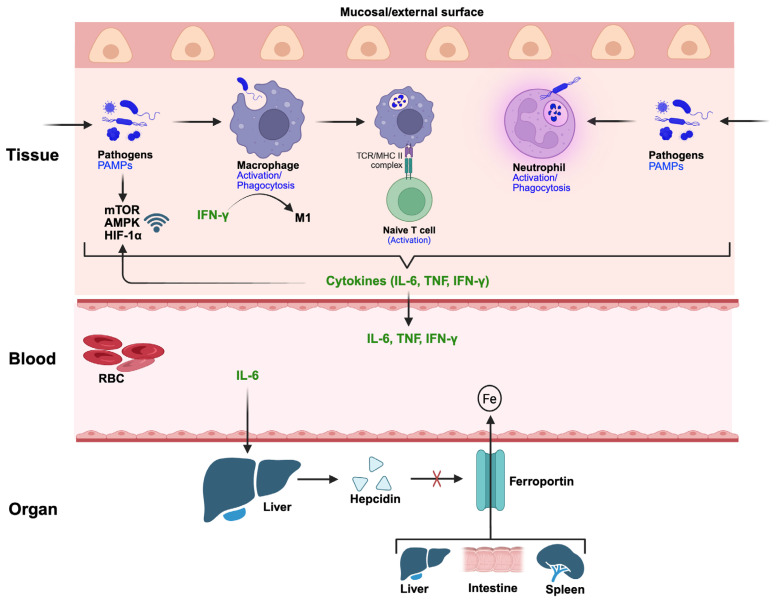
Illustration of immune sensing and activation of restrictive immunometabolism. Upon infection, pathogens trigger the immune system and repressive immunometabolic pathways directly or through pathogen associated molecular patterns (PAMPs). The pathogen derived signals and inflammatory mediators activate innate immune cells like macrophage and neutrophils. These cells produce cytokines like interleukin-6 (IL-6), tumor necrosis factor (TNF), and interferon gamma (IFN-γ). The activated innate immune cells like macrophage and neutrophil will contend with the pathogens but the macrophage also activate the adaptive immune cells like T-cells via antigen presentation using the major histocompatibility complex II (MHCII) and T cell receptor (TCR). The innate and adaptive immune cells collectively produce more cytokines. The engagement of pathogen receptors alongside the produced cytokines reprograms local and systemic metabolism to limit nutrient availability to invading pathogens. For example, IFN-γ induces the activation of macrophages to the M1 phenotype which restricts or toxifies invading pathogens with nutrients. Also, IL-6 trigger hepcidin expression by the liver which in turn downregulates ferroportin-facilitated export of iron from storage organs like liver, intestine, and spleen. The activation of the immune system via pathogen signals also favors the modulation of signaling pathways like rapamycin (mTOR), AMP-activated protein kinase (AMPK), and hypoxia-inducible factor-1α (HIF-1α) in ways that favors nutrient restriction from the invading pathogen.

The activation of repressive immunometabolic pathways during infection is initiated by a multilayered sensing network that integrates pathogen-derived signals, host-derived danger cues, and systemic inflammatory mediators. Figure 6 illustrates how the immune system senses pathogen invasion, consequently leading to the activation of repressive immunometabolism. At barrier sites and within infected tissues, immune and epithelial cells detect conserved microbial structures, pathogen-associated molecular patterns (PAMPs), through pattern recognition receptors (PRRs), including Toll-like receptors (TLRs), NOD-like receptors (NLRs), and C-type lectin receptors [223,224]. Engagement of these receptors rapidly activates transcriptional programs that rewire cellular metabolism, favoring antimicrobial effector functions while restricting nutrient availability to invading pathogens [25,225]. These early sensing events represent the primary trigger linking immune recognition to nutrient restriction. A central communication axis connecting innate immune sensing to repressive immunometabolism is cytokine signaling. Pro-inflammatory cytokines such as interleukin-6 (IL-6), tumor necrosis factor (TNF), and interferon-γ (IFN-γ) act locally and systemically to reshape host nutrient handling. For example, IL-6 induces hepatic hepcidin expression, which suppresses ferroportin-mediated iron export from macrophages, splenocytes, enterocytes, and hepatocytes, thereby enforcing hypoferremia of inflammation [11,226]. Also, IFN-γ drives macrophage activation toward a classically activated (M1) state characterized by enhanced glycolysis, reduced mitochondrial respiration, and increased sequestration of iron, zinc, and manganese within intracellular compartments inaccessible to pathogens [7,227]. These cytokine-mediated signals ensure that metabolic restriction is coordinated at both cellular and organismal scales. In parallel, immune cells integrate metabolic stress and nutrient availability signals through intracellular nutrient-sensing pathways. Key regulators such as mammalian target of rapamycin (mTOR) [228], AMP-activated protein kinase (AMPK) [229], and hypoxia-inducible factor-1α (HIF-1α) [230,231] function as molecular hubs linking immune activation to metabolic reprogramming. Pathogen recognition and inflammatory cytokines suppress mTOR signaling in specific contexts, promoting autophagy and limiting anabolic processes that would otherwise support pathogen replication [232]. Conversely, AMPK activation during energetic stress reinforces catabolic pathways and supports iron and lipid sequestration [233]. HIF-1α stabilization, often induced by inflammatory signaling even under normoxic conditions, enhances glycolytic flux while repressing oxidative metabolism, indirectly restricting metabolites exploited by intracellular pathogens [234]. Damage-associated molecular patterns (DAMPs) generated during tissue injury further amplify repressive immunometabolic signaling. Molecules such as extracellular ATP, high mobility group box 1 (HMGB1), and oxidized lipids signal through purinergic receptors and inflammasome pathways to reinforce nutrient restriction and antimicrobial metabolism [235]. Inflammasome activation, particularly via NLR family pyrin domain containing 3 (NLRP3), not only promotes IL-1β secretion but also alters cellular iron and zinc trafficking, enhances ferritin expression, and promotes metal sequestration within lysosomal and phagosomal compartments [65]. Thus, metabolic repression emerges not only from pathogen detection but also from host-derived indicators of cellular stress and damage. Finally, systemic neuroendocrine and hormonal signals integrate immune activation with whole-body metabolic adaptation. For instance, acute-phase responses initiated by inflammatory cytokines remodel hepatic protein synthesis, shifting production toward metal-binding and transport proteins such as ferritin, ceruloplasmin, and haptoglobin, while downregulating negative acute-phase proteins like albumin that normally circulate nutrients [236]. Concurrently, glucocorticoids and stress hormones modulate immune cell metabolism, tempering excessive inflammation while maintaining nutrient restriction [237]. Together, these layered communication pathways ensure that repressive immunometabolism is activated and precisely tuned to infection severity, pathogen niche, and tissue context.

### 4.2. Regulation of Nutrient Availability at Sites of Infection

Following immune sensing and activation of repressive immunometabolism, the host does not necessarily orchestrate a passive depletion of nutrients. Rather, the host actively redistributes essential micronutrients to restrict microbial access while preserving host viability [21]. At sites of infection, including mucosal surfaces like the intestine, the host actively compensates for microbial nutrient demand through coordinated systemic and cell-intrinsic regulatory mechanisms that alter concentrations without necessarily altering total body abundance. At the systemic level, inflammatory cytokines, most prominently IL-6, IL-1β, TNF, and IFN-γ, reprogram nutrient homeostasis by acting on endocrine organs and the liver [238,239]. A canonical example is IL-6-driven induction of hepcidin, which promotes internalization and degradation of the iron exporter ferroportin, thereby decreasing dietary iron absorption and trapping iron within storage sites [11]. Similar cytokine-dependent mechanisms govern zinc and copper distribution by modulating the expression of metallothioneins and metal transporters, underscoring the endocrine nature of nutritional immunity [240]. At the tissue level, infection induces profound remodeling of epithelial and stromal compartments that alters nutrient gradients. In the intestine, inflammatory signaling suppresses expression of absorptive transporters for iron and zinc while enhancing secretion of metal-binding proteins such as lipocalin-2, which sequesters siderophore-bound iron in the lumen [14]. At sites of localized infection, vascular permeability and extracellular matrix remodeling further influence micronutrient diffusion and availability, creating microenvironments that are hostile to pathogens but metabolically demanding for host cells [241]. Within immune and epithelial cells, nutrient concentrations are tightly controlled through coordinated regulation of transporters, storage proteins, and intracellular trafficking pathways. For example, macrophages and neutrophils upregulate ferritin to sequester iron, activate NRAMP1 to export divalent metals from phagosomes, and modulate ZIP/ZnT transporter expression to dynamically adjust cytosolic zinc pools [242,243]. These changes are tightly coupled to antimicrobial effector functions, as metal availability directly influences oxidative burst capacity, enzyme activity, and inflammasome signaling. Finally, infection-associated changes in nutrient concentrations are inseparable from immunometabolic reprogramming. For instance, activated immune cells undergo a metabolic shift toward aerobic glycolysis, altered amino acid utilization, and lipid remodeling, all of which reshape intracellular nutrient demand and competition with pathogens [25]. Thus, nutrient regulation during infection reflects a dynamic equilibrium between host defense, immune cell bioenergetics, and tissue homeostasis rather than a unidirectional depletion process.

### 4.3. Baseline Versus Infection-Induced Nutrient Levels

Under homeostatic conditions, circulating and tissue nutrient concentrations are maintained within narrow physiological ranges through tightly regulated absorption, storage, and recycling mechanisms. For instance, iron is largely sequestered within hemoglobin, ferritin, and transferrin, leaving only a small, highly regulated plasma pool that minimizes oxidative toxicity while ensuring erythropoiesis [244]. Zinc, copper, and manganese similarly exist predominantly in protein-bound forms, reflecting the dual necessity of bioavailability and toxicity prevention. Baseline nutrient values, however, provide limited insight into the microenvironments encountered by pathogens during infection. While systemic iron concentrations may decline modestly during inflammation, the effective iron availability at infection sites can fall precipitously due to localized sequestration and transporter reprogramming [21]. This distinction highlights a critical conceptual point, that nutritional immunity operates primarily through spatial redistribution, not global deficiency. During infection, rapid-onset hypoferremia of inflammation can decrease serum iron levels by more than half within hours, driven by hepcidin-mediated ferroportin degradation [226]. Concurrently, ferritin levels rise, reflecting iron sequestration rather than increased iron load. Similar patterns are observed for zinc, where plasma concentrations decline during acute infection as zinc is redistributed into the liver and immune cells, despite unchanged total body zinc [191]. At the tissue and cellular levels, infection-induced nutrient shifts are even more pronounced. For instance, phagosomes within activated macrophages become profoundly depleted of iron and manganese while accumulating copper and zinc, creating a chemically hostile environment for engulfed microbes [9]. These compartment-specific changes are invisible to systemic measurements yet critically determine infection outcomes. Importantly, prolonged or dysregulated infection-induced nutrient redistribution can transition from protective to pathological. For example, chronic hypoferremia contributes to anemia of inflammation, while sustained zinc sequestration impairs epithelial repair and adaptive immune responses [11,245]. Thus, infection-induced deviations from baseline nutrient levels represent a double-edged sword, balancing antimicrobial efficacy against host physiological cost.

### 4.4. Trace Elements Altered Across Infection Types

Different classes of pathogens elicit distinct trace-element signatures that reflect both host defense strategies and pathogen-specific vulnerabilities. For example, bacterial infections typically induce robust sequestration of iron, zinc, and manganese, metals essential for bacterial metabolism and antioxidant defenses, while simultaneously increasing copper concentrations within phagolysosomes as a direct antimicrobial stressor [21]. Intracellular bacterial pathogens such as *Mycobacterium tuberculosis* and *Salmonella enterica* are particularly sensitive to phagosomal metal restriction [5,165]. Host cells limit manganese and iron availability to impair superoxide dismutase activity and respiratory metabolism, while copper and zinc intoxication further destabilizes bacterial proteins and membranes [43,246]. By contrast, viral infections exert more complex and indirect effects on trace-element homeostasis. For example, many viruses induce ferritin expression and alter iron trafficking to support replication while simultaneously triggering inflammatory hypoferremia [14]. Zinc availability is also frequently perturbed, as zinc-dependent transcription factors and antiviral enzymes are sensitive to fluctuations in intracellular zinc pools [193]. Fungal pathogens encounter strong zinc and iron limitation during infection, particularly at mucosal surfaces. For instance, host proteins such as calprotectin impose severe zinc and manganese restriction, forcing fungi to deploy high-affinity acquisition systems [247]. These interactions have revealed trace elements as critical determinants of fungal virulence and niche specificity. Parasitic infections introduce additional complexity, as helminths and protozoa can both induce and exploit nutrient redistribution. Some parasites benefit from iron-rich environments, while others trigger host iron-withholding responses that exacerbate anemia and immune suppression [248]. Together, these examples underscore that trace-element dynamics are infection-type specific, shaped by pathogen biology, tissue tropism, and immune context.

### 4.5. Impact of Nutrient Deficiency on Innate and Cellular Immunity

Deficiencies in trace elements, proteins, or vitamins remarkably alter innate and cellular immune responses by disrupting signaling pathways, effector mechanisms, and cellular development. For instance, iron deficiency impairs macrophage antimicrobial activity by lowering reactive oxygen species generation and nitric oxide synthesis, while excess iron promotes pathogen growth and suppresses immune activation [244]. Zinc deficiency exerts particularly broad immunological effects. Because zinc is required for NF-κB signaling, DNA replication, and antioxidant defense, its deficiency compromises epithelial barrier integrity, reduces neutrophil chemotaxis, and impairs natural killer cell cytotoxicity [245]. These defects translate into increased susceptibility to bacterial, viral, and fungal infections. Vitamins play equally critical roles in immune competence. For example, vitamin A deficiency disrupts mucosal immunity by impairing epithelial differentiation and IgA production [249], while vitamin D deficiency reduces antimicrobial peptide expression and macrophage activation [250]. B-vitamin deficiencies further compromise lymphocyte proliferation and metabolic flexibility [251]. Protein deficiency, often accompanying micronutrient scarcity, exacerbates immune dysfunction by limiting amino acid availability for cytokine synthesis and clonal expansion [133,252]. These combined deficiencies are particularly relevant in chronic infections and low-resource settings, where nutritional immunity intersects with malnutrition [253,254,255]. Importantly, immune responses themselves can exacerbate nutrient deficiency. For example, prolonged inflammatory sequestration of iron and zinc may suppress adaptive immunity, creating a feedback loop that impairs pathogen clearance and promotes chronic disease [256]. Thus, nutrient deficiency is not merely a predisposing factor but also a consequence of sustained immune activation.

### 4.6. Immune Cell Population Dynamics Under Nutrient Stress

Nutrient availability directly influences immune cell development, differentiation, and survival. For instance, zinc and iron deficiencies decrease thymic output, leading to reduced naïve T-cell populations and impaired adaptive immune responses [257]. These effects are especially pronounced during early life and aging, periods characterized by heightened nutritional sensitivity [258,259]. Macrophage populations exhibit functional plasticity in response to nutrient stress. For example, iron-restricted macrophages adopt a pro-inflammatory phenotype optimized for pathogen containment, whereas iron-loaded macrophages display impaired antimicrobial activity and increased permissiveness to intracellular pathogens [7]. Similarly, zinc availability shapes macrophage polarization and cytokine production, with zinc deficiency often promoting M1 polarization and pro-inflammatory cytokines (like TNF-α, IL-1β, IL-6) via NF-κB, worsening inflammation, while zinc supplementation can shift them towards the M2 phenotype, reducing inflammation and promoting tissue healing [260,261]. Neutrophils are acutely sensitive to trace-element fluxes. For example, zinc and copper regulate granule composition, oxidative burst capacity, and the activity of calprotectin, a key antimicrobial protein that enforces metal starvation [257,262,263]. Deficiency of zinc compromises neutrophil killing efficiency and increases susceptibility to extracellular infections [257]. Lymphocyte responses are tightly coupled to vitamin and mineral availability. For instance, vitamins A and D influence T-helper cell differentiation, regulatory T-cell function, and memory formation [264,265], while iron and zinc support proliferation and effector differentiation [84,266,267]. Chelation therapies or excessive endogenous chelators can therefore inadvertently suppress adaptive immunity. Collectively, these findings highlight that immune cell population dynamics are not fixed but nutrient-responsive, with profound implications for infection outcomes, vaccine efficacy, and therapeutic interventions targeting nutritional immunity.

## 5. Therapeutic Implications

Nutritional immunity not only serves as a major pillar for host defense but also presents a fertile landscape for therapeutic innovation. Through the comprehension and manipulation of nutritional immunity dynamics, researchers and clinicians can develop targeted interventions that either starve pathogens, strengthen host defense strategies, or weaken pathogens’ counterstrategies. Therapeutic strategies inspired by nutritional immunity, including their mechanisms of action, examples, clinical trials, challenges, and translational outlook, are presented in Table 3. Overall, this section evaluates the translational potential of nutritional immunity across antimicrobial strategies, host-directed therapies, and diagnostic applications.

### 5.1. Targeting Nutrient Availability for Infection Control

Manipulating the availability of nutrients is a promising approach for controlling infections, particularly in an era of rising antimicrobial resistance. Iron chelation, for example, has been considered as a strategy to restrict microbial growth by depriving pathogens of this critical cofactor. Agents such as deferoxamine and deferiprone bind free iron, lowering its bioavailability and thereby hindering the proliferation of iron-dependent pathogens like *Staphylococcus aureus* and *Escherichia coli* [315]. However, therapeutic iron chelation must be delicately balanced to avoid exacerbating anemia or impairing host immunity [7]. Beyond iron, zinc and manganese sequestration have emerged as viable antimicrobial strategies. For instance, calprotectin, a host protein that chelates these metals, has inspired the development of synthetic mimetics that mimic calprotectin’s metal-binding properties [316]. These agents can be deployed locally at infection sites to increase metal starvation without systemic toxicity [72]. Moreover, targeting microbial metal transporters with small-molecule inhibitors offers a pathogen-specific approach that circumvents traditional antibiotic mechanisms [317,318]. Carbon source restriction is another avenue for controlling infection. By manipulating host metabolism or blocking microbial access to key substrates, such as glucose or fatty acids, researchers can create unfavorable environments for pathogens. For instance, inhibitors of bacterial glycerol uptake have demonstrated efficacy against *Listeria monocytogenes*, which depends on host-derived glycerol during intracellular growth [319]. These strategies underscore the potential of metabolic interference as a non-antibiotic antimicrobial approach. Nevertheless, targeting nutrient availability must factor in host–pathogen specificity and tissue context. This is because nutrient manipulation may have inadvertent implications on host cells, particularly immune cells that depend on similar substrates for their activation and function. Thus, precision targeting, guided by pathogen biology and host physiology, is critical for the safe and effective deployment of nutrient-based therapies.

### 5.2. Novel Therapeutic Strategies Based on Nutritional Immunity

Recent advances in molecular biology and immunology have enabled the design of novel therapeutics that exploit or mimic nutritional immunity. One example of such a strategy involves the development of siderophore–antibiotic conjugates, also known as “Trojan horse” antibiotics. These compounds utilize bacterial iron uptake systems to deliver antibiotics directly into the pathogen. For instance, cefiderocol, a siderophore–cephalosporin conjugate, has shown potent activity against multidrug-resistant Gram-negative bacteria by seizing iron transport pathways [320]. Another innovative approach involves the use of engineered probiotics that battle with pathogens for nutrients. These beneficial microbes can be designed to sequester iron, zinc, or amino acids in the gut, thereby impeding pathogen colonization. Advances in synthetic biology are enabling the rational design of probiotic strains with enhanced nutrient-scavenging capabilities, positioning the microbiome as an active component of host defense. For instance, *Lactobacillus* strains engineered to express heterologous siderophore receptors or metal-binding proteins have demonstrated the ability to reduce intestinal colonization by pathogens such as *Salmonella enterica* in animal models [321,322]. These engineered microbes function by intercepting pathogen-accessible nutrients, effectively extending host nutritional immunity into the microbial community. Such strategies illustrate the therapeutic potential of microbiome-based interventions that leverage nutrient competition to prevent or mitigate infection, particularly in settings where antibiotic use is undesirable or ineffective. Also, standard antibiotics combined with nutrient iron chelators have shown amplified antimicrobial activity. For instance, the impregnation of catheters with both antimicrobial agents and iron chelators gave increased antimicrobial and anti-biofilm activity than just antimicrobial agents alone [69]. Also, thiostrepton, a Gram-positive thiopeptide antibiotic imported via pyoverdine receptors, synergized with the iron chelator deferasirox to yield higher growth suppression for *P. aeruginosa* and *Acinetobacter baumannii* in clinical isolates than thiostrepton alone [323]. Further, nanotechnology finds application in nutritional immunity-based therapeutics. For example, nanoparticles functionalized with metal-binding ligands can be employed to sequester iron or zinc at infection sites, creating localized nutrient deserts [324]. These platforms can be further adjusted to deliver immunomodulatory agents or antibiotics, enhancing their therapeutic efficacy [325,326]. The modularity of nanomedicine permits tailored interventions based on infection site and pathogen type. Essentially, these novel strategies must be evaluated for their impact on host nutrient homeostasis and immune function. While mimicking nutritional immunity can improve pathogen clearance, excessive nutrient deprivation may impair tissue repair or compromise immune activation. Thus, therapeutic design must integrate insights from host–pathogen interactions, nutrient biology, and immunometabolism to ensure the best outcomes.

### 5.3. Nutrient-Based Therapies for Specific Infections

Nutrient-based therapies exploit the reliance of pathogens on host-derived metabolites and micronutrients, transforming principles of nutritional immunity into targeted therapeutic interventions. Unlike conventional antimicrobials that directly inhibit microbial viability, these approaches aim to reshape the metabolic landscape of infection, limiting pathogen access to critical nutrients while preserving or even enhancing host immune function. Such strategies are particularly attractive in infections where nutrient acquisition is tightly coupled to virulence, persistence, or immune evasion. However, the success of nutrient-based interventions depends on a nuanced understanding of pathogen metabolic requirements, host nutritional status, and infection-associated immunometabolic remodeling [7,21,25]. Malaria represents a paradigmatic example of nutrient-targeted therapy. For instance, *Plasmodium falciparum* depends on the digestion of host hemoglobin within the food vacuole to obtain amino acids and iron, generating toxic heme as a byproduct that must be detoxified for parasite survival [178,179]. Host iron status strongly influences malaria severity, transmission, and treatment outcomes, with iron supplementation increasing susceptibility in endemic regions [327,328]. Iron chelation strategies using deferoxamine and related compounds have therefore been explored to restrict parasite growth by limiting iron availability. While some clinical and experimental studies demonstrate antiparasitic effects, others highlight the risks of host iron depletion, anemia, and impaired immune competence, underscoring the need for careful patient stratification [329,330,331]. These findings illustrate both the promise and limitations of nutrient deprivation as a therapeutic modality. In tuberculosis, nutrient-based therapies intersect with the pathogen’s dependence on host iron and lipids for intracellular persistence. *Mycobacterium tuberculosis* adapts to the nutrient-restricted environment of macrophages by scavenging iron through siderophores and exploiting host-derived fatty acids and cholesterol as carbon sources [332]. Also, host-directed strategies that modulate iron trafficking or lipid metabolism have emerged as attractive adjuncts to antituberculosis therapy. For example, pharmacological manipulation of host iron sequestration pathways may restrict bacterial replication, while targeting lipid availability can disrupt the metabolic flexibility required for long-term persistence [333]. Statins, which alter host cholesterol biosynthesis and exert immunomodulatory effects, have been shown to enhance phagosome maturation, improve antimicrobial responses, and reduce mycobacterial burden in experimental models [334]. These findings highlight how repurposed metabolic drugs can synergize with nutritional immunity. Fungal infections further illustrate the therapeutic potential of nutrient manipulation. Pathogens such as *Candida albicans* and *Cryptococcus neoformans* depend heavily on iron acquisition for growth, morphogenesis, and virulence, employing high-affinity uptake systems to overcome host sequestration mechanisms [335,336]. Iron chelation and iron-binding compounds have demonstrated efficacy in reducing fungal burden in animal models and are being explored as adjunctive therapies to enhance antifungal efficacy [268,337,338]. Such approaches are particularly relevant in immunocompromised populations, including transplant recipients and critically ill patients, where invasive fungal infections are difficult to eradicate and resistance to standard antifungals is increasing [339]. Beyond iron, emerging nutrient-based therapies target other metabolic dependencies, including zinc, manganese, amino acids, and vitamins. For instance, limiting zinc availability can impair bacterial enzyme function and stress resistance [340,341], whereas vitamin manipulation, such as vitamin D supplementation, may enhance antimicrobial peptide production and macrophage function in select infections [342,343]. However, these strategies must be approached cautiously, as excessive chelation or deficiency can compromise host immunity, tissue repair, and hematopoiesis [11,257]. Consequently, nutrient-based therapies should not be viewed as universally restrictive but rather as precision interventions tailored to infection stage, pathogen niche, and host nutritional and inflammatory status. Ultimately, the therapeutic exploitation of nutritional immunity requires personalized and systems-level approaches. Integrating host nutritional biomarkers, pathogen metabolic profiling, and immunometabolic state will be essential for identifying patients who are most likely to benefit from nutrient-based interventions while avoiding harm. Advances in metabolomics, systems biology, and host-directed therapy design offer promising avenues to refine these strategies, positioning nutrient modulation as a powerful adjunct to antimicrobial treatment rather than a standalone solution [25,227].

### 5.4. Host-Directed Therapies to Enhance Nutritional Immunity

Host-directed therapies (HDTs) aim to strengthen the host’s natural defenses, including nutritional immunity, to fight infection. One strategy involves increasing the expression or activity of nutrient-sequestering proteins such as calprotectin, hepcidin, and lipocalin-2. For example, synthetic hepcidin analogs have been developed to lower serum iron levels and repress bacterial growth in sepsis models [11]. These agents mimic the host’s iron-chelating response and may serve as supplements to antibiotic therapy. Immunomodulatory agents can also be used to switch on pathways that limit nutrient availability. Interferon-γ, for instance, triggers indoleamine 2,3-dioxygenase (IDO), which depletes tryptophan and restrains intracellular pathogens like *Toxoplasma gondii* and *Chlamydia trachomatis* [135]. Enhancing IDO activity or mimicking its effects may present therapeutic benefits in chronic infections where immune evasion is common. Further, gene therapy and RNA-based approaches are emerging tools for controlling host nutrient responses. CRISPR-based editing of genes involved in iron metabolism or metal transport could also be used to improve resistance to specific pathogens. Similarly, siRNA targeting of host nutrient transporters may lower pathogen access to critical substrates without impairing host function [344]. These technologies provide precision control over host–pathogen interactions. Despite their potential, HDTs must be carefully examined for off-target effects and long-term safety. Modulating nutrient availability can affect immune cell function, tissue repair, and systemic metabolism [345]. Therefore, HDTs should be integrated into a broader therapeutic framework that considers nutritional status, pathogen biology, and host immunity.

### 5.5. Diagnostic Biomarkers of Nutritional Immunity

The active interplay between host nutrient sequestration and pathogen adaptation produces quantifiable changes in biomarker profiles that can be explored for diagnostics. The levels of ferritin, transferrin, and hepcidin saturation in the serum are commonly used to determine iron status and inflammation. For example, elevated ferritin and hepcidin levels are suggestive of iron sequestration during infection and can help differentiate between iron-deficiency anemia and anemia of inflammation [11]. Calprotectin, a manganese- and zinc-binding protein, is a well-established biomarker of neutrophil activation and mucosal inflammation [346,347]. Fecal calprotectin is commonly used to monitor inflammatory bowel disease, and its elevation in systemic infections may indicate the activation of nutritional immunity [348]. Similarly, the serum levels of lipocalin-2 may correlate with bacterial infection and may serve as a marker of iron-chelating responses [349]. Metabolomic profiling presents a high-resolution view of nutrient flux during infection. Changes in amino acid levels, such as tryptophan depletion and kynurenine accumulation, portray IDO activity and immune modulation. These metabolites can be measured using mass spectrometry and integrated into diagnostic algorithms for infections like HIV and tuberculosis [135]. Such approaches allow real-time monitoring of host–pathogen interactions. Emerging technologies such as biosensors and wearable devices may further improve the utility of nutritional immunity biomarkers. By continuously tracking nutrient-related parameters, clinicians can personalize therapy and assess the efficacy of treatment. Ultimately, merging biomarker data with genomic and immunologic profiles will facilitate precision diagnostics and targeted therapeutic interventions.

## 6. Future Directions

As the field of nutritional immunity evolves, it is increasingly clear that nutrient sequestration is not merely a passive defense but a dynamic and integrative component of host–pathogen interactions. Future efforts and research must address the mechanistic complexity, translational potential, and global significance of nutritional immunity, particularly in the context of emerging infectious diseases, malnutrition, and microbiome science. Essentially, this section offers a window into the future of a complete understanding of the components and applications of the concept of nutritional immunity.

### 6.1. Emerging Research Areas in Nutritional Immunity

Recent advances in systems biology and high-resolution analytical technologies are transforming the study of nutritional immunity by allowing precise mapping of nutrient availability, sequestration, and utilization at cellular and tissue scales during infection. For instance, single-cell transcriptomics, spatial proteomics, and metabolomics now enable investigators to resolve how immune and epithelial cell subsets differentially regulate metal transporters, amino acid metabolism, and nutrient-sensing pathways in infected tissues [350,351]. These approaches have revealed substantial heterogeneity in nutritional immune responses, including compartment-specific redistribution of iron, zinc, and manganese within phagosomes, mitochondria, and secretory pathways. Importantly, such spatially resolved insights have direct clinical relevance, as they enable identification of biomarkers reflecting functional nutrient restriction rather than bulk serum concentrations, thereby improving diagnostic precision and therapeutic stratification. A second emerging area involves expanding the scope of nutritional immunity beyond classical micronutrients such as iron, zinc, and manganese to include less-studied trace elements and metabolic cofactors. Elements such as selenium and molybdenum are increasingly recognized for their roles in redox homeostasis, antimicrobial toxicity, and enzymatic regulation during infection [23,352]. For example, just as copper can be mobilized into phagolysosomes to exert antimicrobial pressure [94], selenium availability influences antioxidant defenses and immune cell survival [353,354]. Understanding how hosts dynamically regulate these elements and how pathogens sense and adapt to these constraints may uncover new host-directed therapeutic targets and guide micronutrient supplementation strategies that are context-specific rather than empiric. The integration of nutritional immunity with immunometabolism represents another rapidly advancing frontier with clear translational implications. For instance, immune activation is accompanied by remarkable metabolic reprogramming, including increased uptake of glucose, glutamine, and fatty acids to support effector functions such as cytokine production, phagocytosis, and antimicrobial activity [25,352]. This metabolic rewiring intersects directly with nutritional immunity, as competition for nutrients between host and pathogen shapes infection outcomes. Emerging evidence suggests that manipulating host metabolic pathways such as glycolysis, fatty acid oxidation, or amino acid catabolism can indirectly restrict pathogen access to essential nutrients while enhancing immune efficacy [4,355,356,357]. These insights support the development of host-directed therapies that exploit immunometabolic checkpoints to reinforce nutritional immunity without directly targeting microbial viability. Finally, while nutritional immunity has been most extensively characterized in bacterial and fungal infections, its role in viral infections remains an underexplored yet clinically important area. Viruses such as HIV and SARS-CoV-2 are increasingly recognized to modulate host nutrient pathways, including iron metabolism, amino acid availability, and lipid biosynthesis, in ways that influence immune competence and disease severity [66,358]. Elucidating how antiviral immune responses intersect with nutrient restriction and immunometabolic control may reveal novel biomarkers of disease progression and identify metabolic vulnerabilities that can be therapeutically exploited.

### 6.2. Roadmap for Translating Nutritional Immunity into Therapeutics

**Figure 7:**
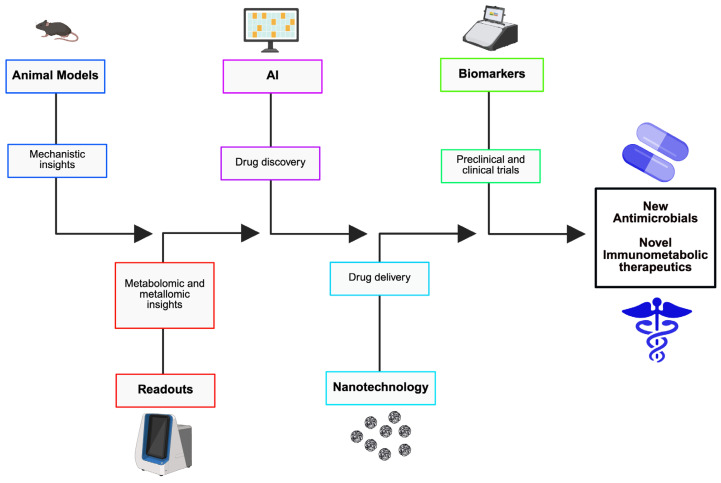
The innovative pipeline that can translate nutritional immunity into therapeutics. The manipulation of nutritional immunity concepts in animal models will present initial mechanistic insights on possible intervention points. Multiomics can then be leveraged to further explore these models and gain informative metabolomics and metallomics readouts. Computational design and artificial intelligence (AI) are integrated to facilitate molecule discovery, design, and screening. Nanocarriers are leveraged for precise, efficient, and controlled delivery of drugs to targets. Robust assessment of nutritional immunity biomarkers in hosts is then used to guide and evaluate preclinical and clinical trials for safety and efficacy of drug candidates, leading to new drugs.

Nutritional immunity presents mechanistic targets for new therapeutics. However, the realization of that potential will be facilitated through the integration of computational design, nanomedicine, and immunometabolic insight. Recent advances in artificial intelligence (AI)-driven molecule discovery, precision nanodelivery, and systems immunometabolism offer a pipeline for agent development that is faster and more targeted than conventional antibiotic screens. Machine-learning and deep-learning platforms have already identified novel antimicrobial scaffolds and optimized siderophore–antibiotic conjugates in silico and in vitro, expediting hit discovery and de-risking medicinal chemistry [359,360]. AI can prioritize compounds that selectively impair pathogen metal acquisition or exploit species-specific uptake systems. Nanocarriers allow for precise spatial control in that they can deliver chelators, metal-mimetic agents (e.g., gallium), or zinc/copper payloads to infected tissues or intracellular vacuoles while limiting systemic toxicity [361,362,363,364]. Functionalization with targeting ligands such as antibodies and peptides can improve pathogen-cell specificity. Host-directed modulation of immune cell metabolism such as altering macrophage metabolism to support nutrient withholding or enhanced antimicrobial effector functions, complements direct antimicrobial strategies and can lower resistance pressure [37,110]. A translational program as illustrated in Figure 7 should (1) pair mechanistic animal models with multiomics readouts, especially metabolomics and metallomics, (2) use AI to triage candidate chemistries that engage pathogen uptake systems, (3) validate targeted nanodelivery in safety models, and (4) design early-phase trials with robust biomarkers, including hepcidin, labile plasma iron, and tissue metallome, to ascertain on-target effects before large efficacy trials [359,361]. This integrated approach promises host-informed, pathogen-targeted therapeutics that exploit nutritional immunity while lowering collateral host injury.

### 6.3. Nutritional Immunity in the Context of Global Health and Malnutrition

Nutritional immunity occupies a critical yet underappreciated position at the intersection of infectious disease biology, nutrition, and global health. In low- and middle-income countries (LMICs), where infectious disease burden and malnutrition frequently coexist, host–pathogen competition for nutrients is profoundly shaped by baseline nutritional status. Iron deficiency anemia, affecting over one-quarter of the global population [365,366], exemplifies this duality. For example, insufficient iron impairs immune effector functions such as oxidative burst and lymphocyte proliferation, yet decreased iron availability can confer partial protection against iron-dependent pathogens, including *Plasmodium falciparum* [14,367]. This evolutionary and epidemiological trade-off complicates public health strategies, as iron supplementation programs may unintentionally increase susceptibility to infection when implemented without infection-sensitive frameworks. Malnutrition fundamentally reshapes the host’s capacity to deploy nutritional immunity mechanisms. For instance, protein-energy malnutrition and micronutrient deficiencies diminish the synthesis and regulation of key host factors involved in nutrient sequestration, including hepcidin, calprotectin, lactoferrin, transferrin, and lipocalin-2 [17,367]. As a result, infected individuals may fail to properly compartmentalize metals and metabolites away from pathogens, weakening both extracellular and intracellular defenses. This creates a self-reinforcing cycle in which infection aggravates nutritional depletion, and nutritional deficiency, in turn, compromises immune-mediated nutrient restriction. Children, pregnant individuals, and immunocompromised populations are particularly vulnerable to this cycle, contributing to disproportionate morbidity and mortality in these groups [368]. From a translational perspective, global health interventions increasingly recognize that nutritional supplementation cannot be decoupled from immune context. For example, beyond correcting deficiency through zinc supplementation, zinc enhances epithelial barrier integrity, modulates innate immune signaling, and reduces the duration and severity of diarrheal disease in children, partly by restoring antimicrobial peptide production and phagocyte function [369,370]. However, blanket supplementation strategies may fail if they do not account for pathogen ecology, inflammatory status, or interactions among micronutrients. Nutritional immunity therefore offers a mechanistic framework for designing context-sensitive interventions that balance immune support with pathogen restriction. Global environmental change further amplifies the relevance of nutritional immunity to public health. For example, climate change, urbanization, food insecurity, and ecosystem disruption are altering patterns of nutrient availability, host nutritional status, and pathogen transmission [371,372]. These shifts may create novel nutrient niches exploited by emerging and re-emerging pathogens, while dietary transitions influence immune resilience at the population level. Integrating nutritional immunity into global health policy thus requires a systems-level approach that accounts for ecological, metabolic, and immunological determinants of infection. Essentially, framing nutritional immunity as a global health principle and not merely a molecular defense mechanism opens new avenues for disease prevention, therapeutic prioritization, and health system resilience.

### 6.4. Nutritional Immunity and the Microbiome

The human microbiome plays a critical role in shaping nutritional immunity. Commensal microbes compete with pathogens for nutrients, generate antimicrobial metabolites, and influence host nutrient metabolism [44,373]. For example, gut bacteria can sequester iron and produce siderophores that inhibit pathogen growth, effectively acting as an extension of the host’s nutritional defense [374,375]. This microbial competition is a major determinant of colonization resistance. The composition of the microbiome also influences host nutrient sensing and immune responses. For example, short-chain fatty acids (SCFAs) synthesized by microbial fermentation of dietary fiber modulate immune cell function and elevate barrier integrity [376,377,378]. These metabolites affect the expression of nutrient transporters and immune regulators, influencing the host’s ability to respond to infection [379]. Dysbiosis, or microbial imbalance, can disrupt these processes and compromise nutritional immunity [380,381]. Probiotic and prebiotic interventions offer a promising strategy to improve nutritional immunity [382]. By promoting beneficial microbes that compete with pathogens and support host nutrient metabolism, these therapies can reinforce immune defenses. For instance, *Lactobacillus* and *Bifidobacterium* strains have been shown to amplify host antimicrobial peptides and modulate iron metabolism, lowering susceptibility to enteric infections [383,384,385]. Such strategies are especially relevant during antibiotic treatment and in vulnerable populations. Future research must illuminate the molecular mechanisms by which the microbiome interacts with nutritional immunity. Integrating microbiome profiling with immunometabolic analysis will essentially provide a holistic view of the interactions between host, pathogen, and microbe. Ultimately, harnessing the microbiome to support nutritional immunity is a frontier in precision medicine and infectious disease prevention.

## 7. Conclusions

Nutritional immunity represents a systems-level defense strategy through which the host reshapes access to macro- and micronutrients to restrain microbial growth, virulence, and persistence. Rather than functioning solely as a collection of nutrient-sequestration mechanisms, nutritional immunity acts as a dynamic regulator of pathogen physiology by imposing metabolic bottlenecks, enforcing trade-offs, and altering the fitness landscapes that pathogens must navigate during infection. In this context, limitation or redistribution of metals, vitamins, amino acids, and carbon sources fundamentally reprograms microbial metabolism and influences evolutionary trajectories, determining infection outcomes across diverse anatomical niches. In turn, pathogens respond to these pressures through adaptive nutrient acquisition systems, metabolic rewiring, and regulatory plasticity, underscoring nutritional immunity as a central arena of host–pathogen conflict. This reciprocal interplay highlights that microbial survival is not dictated by nutrient abundance necessarily, but by access, compartmentalization, and timing within host tissues. Appreciating this distinction reframes infection biology by positioning nutrient availability as a key determinant of microbial behavior rather than a passive consequence of host defense. Importantly, this framework carries substantial translational implications. Therapeutic strategies that manipulate nutrient availability, whether by enhancing host sequestration, disrupting pathogen uptake, or modulating immunometabolism, offer opportunities to complement or circumvent conventional antimicrobial approaches. As antimicrobial resistance continues to rise, targeting nutrient dependencies offers a promising avenue for host-directed therapies, precision nutrition, and microbiome-informed interventions. Ultimately, integrating nutritional immunity into clinical and public health paradigms will be essential for developing sustainable strategies to control infectious diseases in an era shaped by global malnutrition, ecological change, and emerging pathogens.

## Figures and Tables

**Table 1:** Key nutrients in infection at the host–pathogen interface.

S/N	Nutrient	Host Physiological Role	Pathogen Utilization	Pathogen Acquisition Strategies	Infection Context	References
1	Iron (Fe)	Electron transport, oxygen transport, immune cell proliferation.	DNA synthesis, cofactor for respiration, antioxidant enzymes.	Siderophore production (enterobactin, mycobactin), heme uptake systems, transferrin/lactoferrin receptors.	*Mycobacterium tuberculosis*, *Salmonella*, *Escherichia coli*, *Plasmodium falciparum*.	[7,14]
2	Zinc (Zn)	Transcription factors, enzyme catalysis, barrier integrity, innate signaling.	Structural and catalytic cofactor for metabolic enzymes.	High-affinity Zn transporters (ZnuABC), Zn-independent enzymes.	*Staphylococcus aureus*, *Salmonella*, *Candida albicans*	[9,21]
3	Manganese (Mn)	Mitochondrial enzymes, antioxidant defense, immune cell metabolism.	Superoxide dismutase activity, oxidative stress resistance.	Mn importers (MntH, SitABCD), replacement of Fe-dependent enzymes.	*Neisseria*, *Staphylococcus aureus*, *Streptococcus pneumoniae*.	[22]
4	Copper (Cu)	Antimicrobial toxicity in phagosomes, redox reactions.	Respiration and oxidative enzymes (limited tolerance)	Copper efflux pumps (CopA), metallothioneins, Cu-resistant enzymes	*Mycobacterium tuberculosis*, *Salmonella enterica*.	[23]
5	Amino acids (e.g., tryptophan, arginine)	Protein synthesis, immune signaling, nitric oxide production.	Protein synthesis, virulence factor production.	Auxotrophy, host scavenging, altered biosynthetic pathways.	*Chlamydia*, *Toxoplasma gondii*, viruses.	[13,24]
6	Glucose	Primary energy source; fuels immune cell activation.	Glycolysis, biomass generation.	Metabolic flexibility via gluconeogenesis, and use of alternative carbon use.	*Salmonella*, *Listeria monocytogenes*.	[25]
7	Lipids/Cholesterol	Membrane integrity, steroid synthesis, immune signaling.	Carbon source, membrane remodeling, immune evasion.	Host lipid scavenging, lipid droplet hijacking.	*Mycobacterium tuberculosis*, *Chlamydia*, viruses.	[26,27]
8	Vitamins (B complex)	Cofactors for metabolism, immune cell proliferation.	Essential enzyme cofactors	Vitamin biosynthesis or host scavenging	*Salmonella*, gut pathogens.	[28]
9	Biotin (B7)	Fatty acid synthesis, immune metabolism.	Lipid synthesis, virulence regulation.	Biotin scavenging systems, synthesis pathways.	*Mycobacterium tuberculosis*, *Escherichia coli*.	[29,30]

**Table 2:** Summary of nutritional immunity mechanisms.

S/N	Mechanism	Description	Main Host Factors	Targeted Nutrients	Impact on Pathogen	Examples	References
1	Metal sequestration	Host proteins bind essential transition metals firmly, restricting their availability to invading microbes.	Ferritin, lactoferrin, transferrin, hemopexin, haptoglobin.	Fe, Mn, Zn.	Limits cofactor accessibility for bacterial enzymes such as superoxide dismutase and ribonucleotide reductase, thereby impairing replication.	Neutrophil apo-lactoferrin reduces iron accessibility to *Staphylococcus aureus*, calprotectin sequesters zinc and manganese from *Candida albicans*	[5,38,39,40]
2	Metal Toxicity	Host intentionally delivers toxic levels of certain transition metals into pathogen-containing compartments.	Copper transporters (ATP7A, ATP7B), Zinc transporters (ZIP8, ZnT family), NRAMP1 (SLC11A1).	Excess Cu or Zn causing toxicity and/or replacing Fe/Mn.	Overwhelms microbial detoxification systems, causes mis-metalation, and interferes with critical metabolic processes.	Copper toxicity against *Mycobacterium tuberculosis*, Zinc-mediated intoxication of *Streptococcus pneumoniae* and *Mycobacterium tuberculosis*	[41,42,43]
3	Nutrient competition	Host cells outcompete pathogens for limited nutrients in inflamed tissues.	Activated macrophages, neutrophils, T cells.	Glucose, fatty acids, amino acids like tryptophan and arginine.	Stress induced by starvation, forcing bacteria into alternative metabolic pathways or non-replicating forms.	Gut commensals competing with *Salmonella enterica* for trace metals and carbon sources decrease pathogen colonization	[4,44,45]
4	Metabolic reprogramming	Host cells shift metabolic flux to starve pathogens, such as Warburg-like glycolysis, and reduced amino acid availability	HIF-1α, mTOR, autophagy pathways.	Glucose, glutamine, arginine, serine.	Limits access to central carbon and nitrogen sources	Macrophage glycolytic reprogramming restricting *Mycobacterium tuberculosis growth and altering* CCV environment for *Coxiella burnetii*.	[4,46,47]
5	Hormonal regulation	Hormones and inflammatory cytokines regulate systemic nutrient availability.	Hepcidin, TNF-α, IL-6.	Iron via regulation of ferroportin.	Reduces plasma iron, trapping it in macrophages and hepatocytes.	IL-6-induced hepcidin release suppressing iron availability during *Salmonella* infection.	[10,14]
6	Production of antimicrobial peptides (AMPs)	AMPs bind essential metals and exert direct antimicrobial effects.	Psoriasin (S100A7), calprotectin (S100A8/A9), defensins.	Zn, Mn.	Blocks metalloenzyme activity, triggers oxidative stress.	Calprotectin limiting *Staphylococcus aureus* growth in neutrophil extracellular traps (NETs).	[38,48,49]
7	Siderophore interference	Host proteins arrest or neutralize bacterial siderophores to block iron retrieval.	Lipocalin-2 (NGAL), siderocalin.	Iron via siderophore sequestration.	Suppresses iron uptake despite active siderophore production.	Lipocalin-2 binding *Escherichia coli* enterobactin, blocking bacterial iron acquisition.	[50,51,52,53]

**Table 3:** Therapeutic approaches inspired by nutritional immunity.

S/N	Therapeutic Strategy	Mechanism of Action	Examples/Clinical Trials	Challenges	Translational Outlook
1	Iron chelation therapy	Starves pathogens of iron by binding free iron, mimicking host nutritional immunity.	Deferasirox and its derivatives used against *Candida albicans*, *Candida glabrata*, and *Cryptococcus neoformans* [268,269]; Deferiprone as an adjunctive therapy against *Mycobacterium abscessus* [270,271]; Lactoferrin supplementation in trials studies for neonatal sepsis [272,273].	Risk of host iron depletion, impaired immune function, and anemia.	Early/late clinical (iron chelators already approved for other indications; infection-specific use experimental).
2	Metal supplementation to cause toxicity	Deliver toxic levels of metals such as zinc or copper into pathogen-containing compartments	Zinc oxide nanoparticles for antibacterial activity against *Staphylococcus aureus*, *Escherichia coli*, *Pseudomonas aeruginosa*, and *Bacillus subtilis* [100,101,102]; Copper ionophores such as disulfiram, elesclomol studied for antimicrobial activity against *Mycobacterium tuberculosis*, *Chlamydia trachomatis* and *Neisseria gonorrhoeae* [274,275,276]	Balancing pathogen killing with minimal host toxicity	Preclinical/early clinical.
3	Siderophore decoys and mimics	Neutralize bacterial siderophores or prevent their uptake by pathogens	Gallium potency against chronic *Pseudomonas aeruginosa* airway infections in clinical trials [277]; Lipocalin-2 facilitating host defense against *Klebsiella pneumoniae* [278] and *Escherichia coli* [279]	Toxicity at high doses and pathogen adaptation	Early clinical (gallium); other decoy approaches preclinical.
4	Siderophore–antibiotic conjugates as “Trojan horse” drugs	Antibiotics fused to siderophores leverage bacterial uptake systems to facilitate intracellular drug delivery	Cefiderocol, an FDA-approved drug against multidrug-resistant Gram-negative bacteria and nosocomial pneumonia and complicated urinary tract infections [280]; siderophore-monobactam (BAL30072) potency against multidrug-resistant *Acinetobacter* and multidrug-resistant Gram-negative bacteria [281,282]; synthetic sideromycin conjugates under development [280].	Resistance through altered siderophore receptors and high cost	FDA approved (cefiderocol); siderophore-monobactam and synthetic sideromycin conjugates in preclinical.
5	Immunometabolic modulation	Remodel host metabolism to limit key nutrients such as glucose, amino acids, and vitamins from pathogens	IDO activators for tryptophan depletion in viral and bacterial infections [283,284]; Methionine restriction shown to limit intestinal barrier dysfunction and inflammation caused by *Salmonella typhimurium* [285]; Vitamin D supplementation enhances therapeutic outcomes in tuberculosis [283,284].	Systemic metabolic adverse effects and pathogen metabolic flexibility	Early clinical (IDO, vitamin D).
6	Probiotic-pathogen competition	Beneficial microbes compete with pathogens for nutrients and synthesize siderophores to limit pathogen growth	*Lactobacillus rhamnosus* GG in clinical trials for treating pediatric infections [286,287]; *Bifidobacterium longum* modulates iron metabolism, reduces pathogen colonization [288]; *Escherichia coli* Nissle 1917 competes for iron and prevents pathogen expansion [289,290].	Variability in microbiome responses and context-dependent efficacy	Late clinical/approved (some strains widely marketed, regulated as supplements).
7	Fecal microbiota transplant (FMT)	Restores gut microbial diversity and competitive exclusion against pathogens, restricting access to critical nutrients	FMT for recurrent *Clostridium difficile* infection in Phase III RCTs showing >85% efficacy [291,292,293,294]; studies indicating FMT efficacy and safety against multidrug-resistant Enterobacteriaceae colonization [295,296,297,298,299,300]; emerging FMT trials in sepsis and systemic infections [301,302].	Regulatory challenges, donor variability, and risk of pathogen transfer.	Late clinical/approved (FDA-approved for recurrent *Clostridium difficile*).
8	Phytobiotics (plant-derived compounds)	Plant-derived polyphenols, flavonoids, and alkaloids chelate metals, impair microbial metabolism, and modulate host immunity	Curcumin anti-*Mycobacterium* activity via iron chelation [303,304]; Quercetin enhances zinc uptake and modulates immune responses [305]; Antimicrobial activity of Epigallocatechin gallate (EGCG) from green tea against *Pseudomonas aeruginosa* and *Escherichia coli* [306].	Bioavailability, limited clinical validation, and variability in plant extracts.	Preclinical/early clinical.
9	Nanotechnology-based nutrient modulation	Engineered nanocarriers deliver metals, chelators, or metabolic modulators directly to infection sites	Iron oxide nanoparticles investigated for iron sequestration and pathogen restriction [307,308]; Antibacterial activity and mechanistic insights of gallium-based nanoparticles [309,310]; Silver nanoparticles as next-generation antimicrobial agents [311,312,313,314].	Safety, targeted delivery, and long-term stability concerns	Preclinical

## Data Availability

No new data were generated or analyzed in this study. All data discussed are available in the cited literature.

## Supplementary Materials

The following supporting information can be downloaded at: https://www.mdpi.com/article/10.3390/pathogens15020176/s1.
