# Supplementary material for: Mechanisms and Therapeutic Potential of Nutritional Immunity
Source: Pathogens. 2026 Feb 5;15(2):176. doi: 10.3390/pathogens15020176 (PMC12943674; doi:10.3390/pathogens15020176)
Supplement: Supplementary file 1 [file pathogens-15-00176-s001.zip › pathogens-4038272-SM.pdf]

Article Title

Mechanisms and Therapeutic Potential of Nutritional Immunity

DOI: https://doi.org/10.3390/pathogens15020176

Article Rationale

Nutritional immunity, a host strategy of restricting microbial access to essential nutrients, has emerged as a central pillar of antimicrobial defense. While classical reviews have focused primarily on metal sequestration, recent discoveries reveal that nutritional immunity encompasses a broader spectrum of metabolic interactions involving not only transition metals but also amino acids, lipids, and vitamins. These mechanisms collectively rewire the metabolic landscape of infection sites, influencing pathogen fitness, virulence, and persistence. Despite its importance, the field remains fragmented, with insights scattered across microbiology, immunometabolism, and systems biology. A unified synthesis that integrates these molecular mechanisms with therapeutic potential is lacking. Given the escalating crisis of antimicrobial resistance and the resurgence of interest in host-directed therapeutics, a comprehensive reappraisal of nutritional immunity is both timely and necessary. This review addresses that gap by offering a multidisciplinary framework that interlinks nutrient restriction, pathogen counterstrategies, and translational opportunities. Our synthesis is structured around four conceptual axes: (1) Mechanistic Foundations of Nutritional Immunity, (2) Pathogen Countermeasures and Evolutionary Adaptation, (3) Immunological Regulation, and (4) Therapeutic and Translational Frontiers. We attempt to bridge the gap between fundamental immunometabolic processes and clinical applications, reframing nutritional immunity as both a defensive and therapeutic paradigm. By integrating insights from infectious disease biology, systems immunology, and biomedical innovation, it provides a conceptual map for future research and drug development. This article is positioned to appeal broadly to *Pathogens* journal's readership, encompassing microbiologists, immunologists, infectious disease specialists, and translational biologists.
